# Kinesin 1 regulates cilia length through an interaction with the Bardet-Biedl syndrome related protein CCDC28B

**DOI:** 10.1038/s41598-018-21329-6

**Published:** 2018-02-14

**Authors:** Rossina Novas, Magdalena Cardenas-Rodriguez, Paola Lepanto, Matías Fabregat, Magela Rodao, María Inés Fariello, Mauricio Ramos, Camila Davison, Gabriela Casanova, Lucía Alfaya, Federico Lecumberry, Gualberto González-Sapienza, Florencia Irigoín, Jose L. Badano

**Affiliations:** 1 Human Molecular Genetics Laboratory, Institut Pasteur de Montevideo, Mataojo 2020, Montevideo, CP11400 Uruguay; 2 Unidad de Microscopía Electrónica, Facultad de Ciencias, Iguá 4225, Montevideo, CP11400 Uruguay; 3 Facultad de Ingeniería, Universidad de la República, Julio Herrera y Reissig 565, Montevideo, CP11300 Uruguay; 4 Bioinformatics Unit, Institut Pasteur de Montevideo, Montevideo, Uruguay; 5 Signal Processing Laboratory, Institut Pasteur de Montevideo, Montevideo, Uruguay; 6 Cátedra de Inmunología, DEPBIO, Facultad de Química, Universidad de la República, Av. Alfredo Navarro 3051, Montevideo, Uruguay; 7 Departamento de Histología y Embriología, Facultad de Medicina, Universidad de la República, Gral. Flores 2125, Montevideo, CP11800 Uruguay

## Abstract

Bardet-Biedl syndrome (BBS) is a ciliopathy characterized by retinal degeneration, obesity, polydactyly, renal disease and mental retardation. CCDC28B is a BBS-associated protein that we have previously shown plays a role in cilia length regulation whereby its depletion results in shortened cilia both in cells and *Danio rerio* (zebrafish). At least part of that role is achieved by its interaction with the mTORC2 component SIN1, but the mechanistic details of this interaction and/or additional functions that CCDC28B might play in the context of cilia remain poorly understood. Here we uncover a novel interaction between CCDC28B and the kinesin 1 molecular motor that is relevant to cilia. CCDC28B interacts with kinesin light chain 1 (KLC1) and the heavy chain KIF5B. Notably, depletion of these kinesin 1 components results in abnormally elongated cilia. Furthermore, through genetic interaction studies we demonstrate that kinesin 1 regulates ciliogenesis through CCDC28B. We show that kinesin 1 regulates the subcellular distribution of CCDC28B, unexpectedly, inhibiting its nuclear accumulation, and a ccdc28b mutant missing a nuclear localization motif fails to rescue the phenotype in zebrafish morphant embryos. Therefore, we uncover a previously unknown role of kinesin 1 in cilia length regulation that relies on the BBS related protein CCDC28B.

## Introduction

Primary cilia play a critical role in the regulation of cellular and tissue homeostasis acting in mechano- and chemo-sensation and paracrine signaling. Several important cascades have been shown to operate through the cilium or depend on the integrity of the organelle for their correct sensing and transduction including Sonic Hedgehog (Shh), transforming growth factor β (TGFβ), platelet-derived growth factor receptor α (PDGFRα) and Wnt^1–4^. The physiological relevance of primary cilia is best supported by the fact that its dysfunction results in a number of clinical manifestations, including retinal degeneration, obesity, cystic kidney disease, central nervous system malformations and skeletal defects, while its complete absence is incompatible with life. Furthermore, several of these phenotypes are now considered hallmarks of an underlying ciliary defect and characterize the group of human disorders known as ciliopathies^5–8^.

CCDC28B was originally identified as a second site modifier of the ciliopathy Bardet-Biedl syndrome (BBS, OMIM: 209900) although its contribution is likely variable among cohorts^9–12^. CCDC28B was shown to interact with a number of BBS proteins and reduced levels of CCDC28B due to a splicing mutation correlated with a more severe presentation of the disease in some families^10^. More recently we have shown that CCDC28B plays a role in cilia length regulation both in cells and *in vivo* in *Danio rerio* (zebrafish)^13,14^. We have shown that CCDC28B modulates cilia length at least in part through its interaction with SIN1, a member of the mTOR complex 2 (mTORC2), but independently of the mTORC2 pathway in a process not completely understood^13^.

Ciliogenesis and ciliary length are tightly regulated and the later varies between different cell types suggesting that it can influence or determine specific ciliary functions. Moreover, both short and abnormally elongated cilia have been associated with cilia dysfunction and the ciliopathies^15,16^. Therefore, dissecting the mechanism by which CCDC28B affects cilia length is critical to understand its role in human disease. Here we demonstrate that CCDC28B associates with the kinesin 1 molecular motor via an interaction with kinesin light chain 1 (KLC1). In the kinesin 1 motor complex the light chains associate with KIF5 heavy chains which are encoded by three genes in mammals: *KIF5A*, *KIF5B* and *KIF5C*. These proteins present a similar motor domain located on the N-ter region (N-kinesins) but distinct cargo binding motifs on their C-ter region^17,18^. We show here that CCDC28B interacts not only with KLC1 but also with KIF5B.

The kinesins are members of a superfamily of proteins (KIFs) that is composed of 15 families (kinesin 1 to kinesin 14B)^17,19^. Importantly, different kinesin motors have been shown to play an important role in cilia formation and cilia length regulation. Heterotrimeric kinesin 2 in mammals and other kinesins in other organisms are a key component of the process of intraflagellar transport (IFT). This motor-based machinery moves cargo, IFT particles, important for the formation and function of the cilium both into and out of the organelle. Kinesin 2 is responsible for anterograde movement (from base to tip) while cytoplasmic dynein mediates the retrograde (tip to base) transport^20,21^. Not surprisingly, affecting either structural or regulatory components of IFT has a direct impact on cilia length^22–25^.

In addition to those involved in IFT, other kinesins are known to play a role in cilia biology and cilia length regulation, linking assembly/disassembly of the organelle with the cell cycle. Some kinesins have been shown to promote cilia shortening by affecting microtubule dynamics either in the intraciliary compartment (axoneme) or at the basal body. The kinesin 13 family members KIF24 and KIF2A both localize to the centrosome/basal bodies and remodel cilia through depolymerization of microtubules^26,27^. Furthermore, KIF2A is activated by the cell cycle regulated Polo-like kinase 1 (PLK1), thus promoting cilia disassembly in proliferating cells^27^. Other kinesins localize inside the cilium and their depletion results in abnormally elongated cilia. For example, KIF7 (kinesin 4) and KIF19A (kinesin 8) organize intra-ciliary compartments and affect the stability of the axoneme and tubulin levels through microtubule depolymerizing activities^28,29^. In addition, and depending on the context and organism, kinesin 13 members have been shown to regulate cilia length either entering the cilium and affecting microtubules in the ciliary compartment or by controlling cytoplasmic microtubule depolymerization and hence the availability of tubulin for cilia formation and maintenance^30–33^. Thus, we reasoned that the CCDC28B-Kinesin 1 interaction could be relevant to further understand the role of the BBS-associated protein in ciliogenesis.

Here we show that both KLC1 and KIF5B are found at the basal body region of cilia and play an inhibitory role on cilia extension whereby their depletion results in abnormally elongated cilia. Thus, our results uncover a previously unknown role for kinesin 1 in cilia length regulation. Importantly, through genetic interaction experiments in cells we show that this activity of kinesin 1 is mediated by CCDC28B. Furthermore, our data indicate that kinesin 1 regulates the subcellular localization of CCDC28B, unexpectedly, affecting the nuclear/cytoplasmic distribution of the protein whereby targeting kinesin 1 results in nuclear accumulation of CCDC28B. Importantly, we show that eliminating residues encompassing a NLS domain in the zebrafish ccdc28b ortholog impairs its capacity to rescue the depletion of ccdc28b in zebrafish. Altogether, our results underscore a novel role for a main molecular motor in cilia length regulation and provide further insight into the function of the Bardet-Biedl associated protein CCDC28B, a critical step to understand its contribution to the pathogenesis of BBS.

## Results

### CCDC28B interacts with the kinesin 1 motor

**Figure 1:**
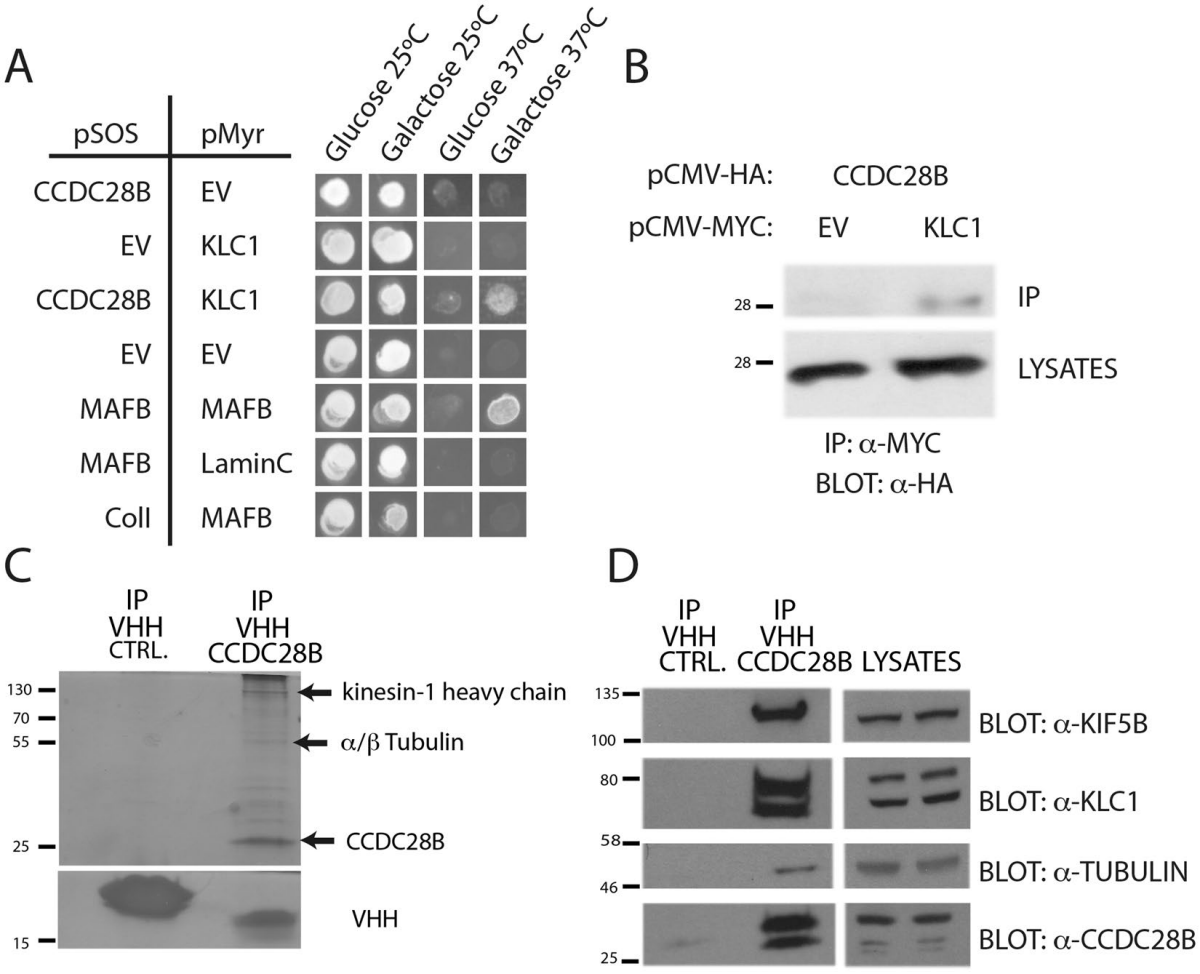
CCDC28B interacts with the kinesin 1 components KLC1 and KIF5B. (**A**) The co-expression of CCDC28B and KLC1 allows *cdc25H* yeast cells to grow at the non-permissive temperature of 37 °C only on galactose that triggers expression from the pMyr construct. Controls: MAFB-MAFB (positive); MAFB-Lamin C (negative), ColI-MAFB (negative), pSOS EV-pMyr EV (negative) and pSOS CCDC28B-pMyr EV (negative). (**B**) HA-CCDC28B is detected only in the Myc-KLC1 immunoprecipitate. Cell lysates are shown to control for protein input. Bands were cropped from the same blot which is shown in Fig. S2A. (**C**) Immunoprecipitation of overexpressed CCDC28B in Hek293 cells with the specific single domain llama antibody (VHH) results in the co-immunoprecipitation of additional proteins. Arrows indicate gel bands analyzed by mass spectrometry. An irrelevant VHH was used as control. The gel was cut in two to avoid saturation of the VHH bands when silver-staining the upper part of the gel. (**D**) The VHH against CCDC28B was used for immunoprecipitation and specific antibodies were used to detect KIF5B, KLC1, α-tubulin and CCDC28B. Cell lysates show the corresponding proteins in the extracts used for immunoprecipitation. Bands shown were cropped from the original blot. The full-length membrane was cut and exposed to the different antibodies (see Fig. S2B,C for blots and for details).

In a cytoplasmic yeast two-hybrid assay, we used full length CCDC28B fussed to the SOS protein (pSOS-CCDC28B) as bait and identified an interaction with kinesin light chain 1 (KLC1) cloned in the pMyr library vector as reported^13^. Temperature sensitive *cdc25h* yeasts were able to grow at the non-permissive temperature of 37 °C only when pMyr-KLC1 was co-expressed with pSOS-CCDC28B but not with the pSOS empty vector (EV) (Fig. 1A). To confirm this interaction we co-expressed HA-tagged CCDC28B and Myc-tagged KLC1 in Hek293 cells and performed a coimmunoprecipitation assay using an anti-Myc antibody. We detected HA-CCDC28B only in the immunoprecipitates from cells co-transfected with Myc-KLC1 but not Myc-EV (Fig. 1B). In addition, we used a single domain llama antibody specific to CCDC28B (VHH; Fig. S1A) to immunoprecipitate the protein from cell lysates of Hek293 transfected with pCS2+ _CCDC28B, we ran the immunoprecipitates in a SDS-PAGE, and silver stained the gel to identify proteins co-immunoprecipitating with CCDC28B. In addition to CCDC28B, we detected proteins at different molecular weights. We cut the main gel bands and analyzed them by mass spectrometry identifying the kinesin 1 heavy chain KIF5B and α/β-tubulin (Fig. 1C; Supplementary Table 1). We confirmed all these interactions by performing immunoprecipitations with the anti-CCDC28B VHH in non-transfected cells to pull down endogenous CCDC28B and western blots using specific antibodies to detect KIF5B, KLC1, α-tubulin and our rabbit polyclonal antibody to detect CCDC28B (Fig. 1D; Fig. S1B; available full-length blots are shown in Fig. S2). Overall, our results indicate that CCDC28B can interact with KLC1 likely in the context of the kinesin 1 molecular motor complex.

### Knockdown of KLC1 and KIF5B in human hTERT-RPE cells result in abnormally elongated cilia

**Figure 2:**
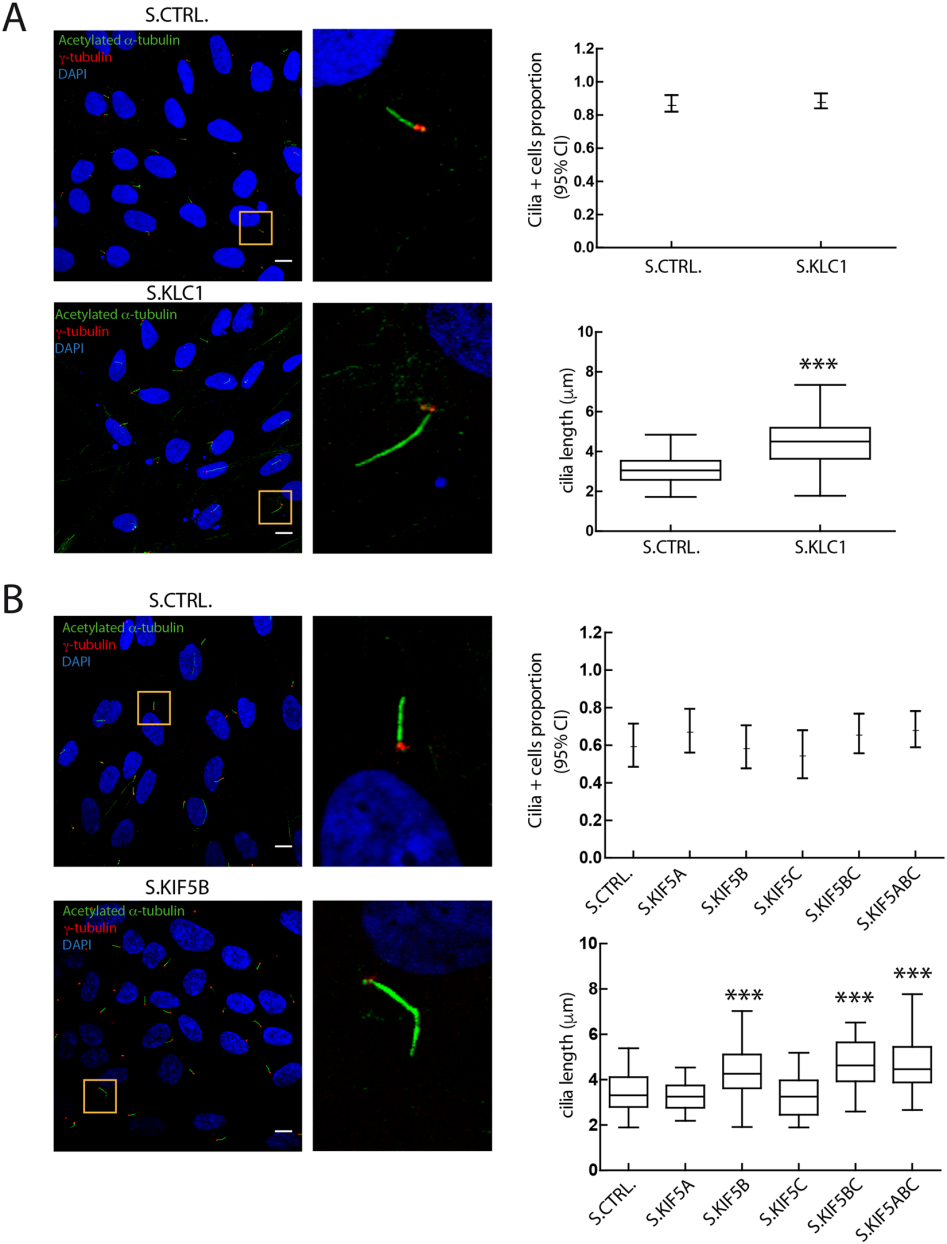
Depletion of KLC1 and KIF5B results in elongated cilia. hTERT-RPE cells were analyzed by confocal microscopy using anti-acetylated tubulin (green), anti γ-tubulin (red) and DAPI (blue) to stain cilia, basal bodies and nuclei respectively. Cilia length was measured and results are expressed as box plots. Results are representative of three independent experiments. (**A**) While knockdown of KLC1 did not affect the proportion of cilia-positive cells compared to a control stealth (hypothesis test for proportions), it did result in significantly elongated cilia (statistical test: Mann-Whitney; ***P < 0.0001). At least 100 cilia were measured per condition (143 for S.Ctrl. and 147 for S.KLC1). (**B**) Knockdown of the different KIF5s (S.KIF5A, B, C) did not affect the proportion of cilia-positive cells. Similarly to KLC1 KD, depletion of KIF5B, KIF5BC and KIF5ABC resulted in elongated cilia (122 KIF5B KD, 96 KIF5BC and 109 KIF5ABC cilia were measured and compared to 95 control). Statistical test: one-way ANOVA. Asterisks denote statistical significant differences compared to controls. ***P < 0.0001. Scale bars correspond to 10 μm.

We have shown previously that depletion of CCDC28B results in shortened cilia in hTERT-RPE cells^14^ and hypothesized that the CCDC28B-kinesin 1 interaction could be relevant to this function. There are three isoforms of KLC1, which only differ in their C-termini (NM_005552.4; NM_182923.3; NM_001130107.1; Fig. S3A). We therefore validated a stealth double stranded RNA oligo (Invitrogen) designed to target all three KLC1 isoforms by RNAi (Fig. S3A,B). We transiently transfected hTERT-RPE cells with our RNA oligos, serum deprived cells for 48 hours to induce ciliogenesis, and assessed cilia 72 hours after transfection using an anti-acetylated α-tubulin antibody and confocal microscopy. We measured both the proportion of cilia-positive cells (cells with a clear axoneme-like acetylated tubulin signal) and cilia length analyzing a minimum of 10 randomly selected fields per condition in three independent experiments. While knockdown (KD) of *KLC1* did not affect the proportion of cilia-positive cells, the median length of cilia was significantly higher than in cells transfected with a control RNA oligo (median of 3.1 μm in controls compared to a median of 4.5 μm in *KLC1* KD cells; *P* = 8.24E-28; Fig. 2A).

Kinesin 1 can be formed by either of three different heavy chains: KIF5A, KIF5B and KIF5C. We obtained RNAi oligos to all three transcripts and tested their efficiency by RT-PCR. Both *KIF5B* and *KIF5C* are expressed in hTERT-RPE cells and transfection of the stealth RNA oligos resulted in significant knockdowns (Fig. S3C,D). In contrast, we were not able to amplify *KIF5A* (Fig. S3C) suggesting that its expression is low in this cell line. Despite this observation, a stealth RNA oligo targeting *KIF5A* was included in the analysis. Similarly to KLC1, KD of the different heavy chains did not largely affect the proportion of cilia-positive cells (Fig. 2B) but depleting KIF5B resulted in a significant elongation of cilia (a median of 3.3 μm in controls vs 4.3 μm in *KIF5B* KD cells; *P* = 2.30E-11). In contrast, the single KD of the other KIF5s was not sufficient to affect cilia length (Fig. 2B). The simultaneous KD of KIF5B and C or KIF5A, B, and C resulted in a subtle exacerbation of the KIF5B phenotype although it was not statistically significant when compared to KIF5B alone (4.6 μm in KIF5BC KD, *P* = 0.74, and 4.5 μm in KIF5ABC KD, *P* = 0.81; Fig. 2B). Thus, our results indicate that while KIF5B KD accounts for the majority of the observed cilia phenotype, a partial functional overlap with other heavy chains, particularly KIF5C, cannot be discarded.

### KLC1 and KIF5B localize at the base of cilia but were not observed in the cilium

**Figure 3:**
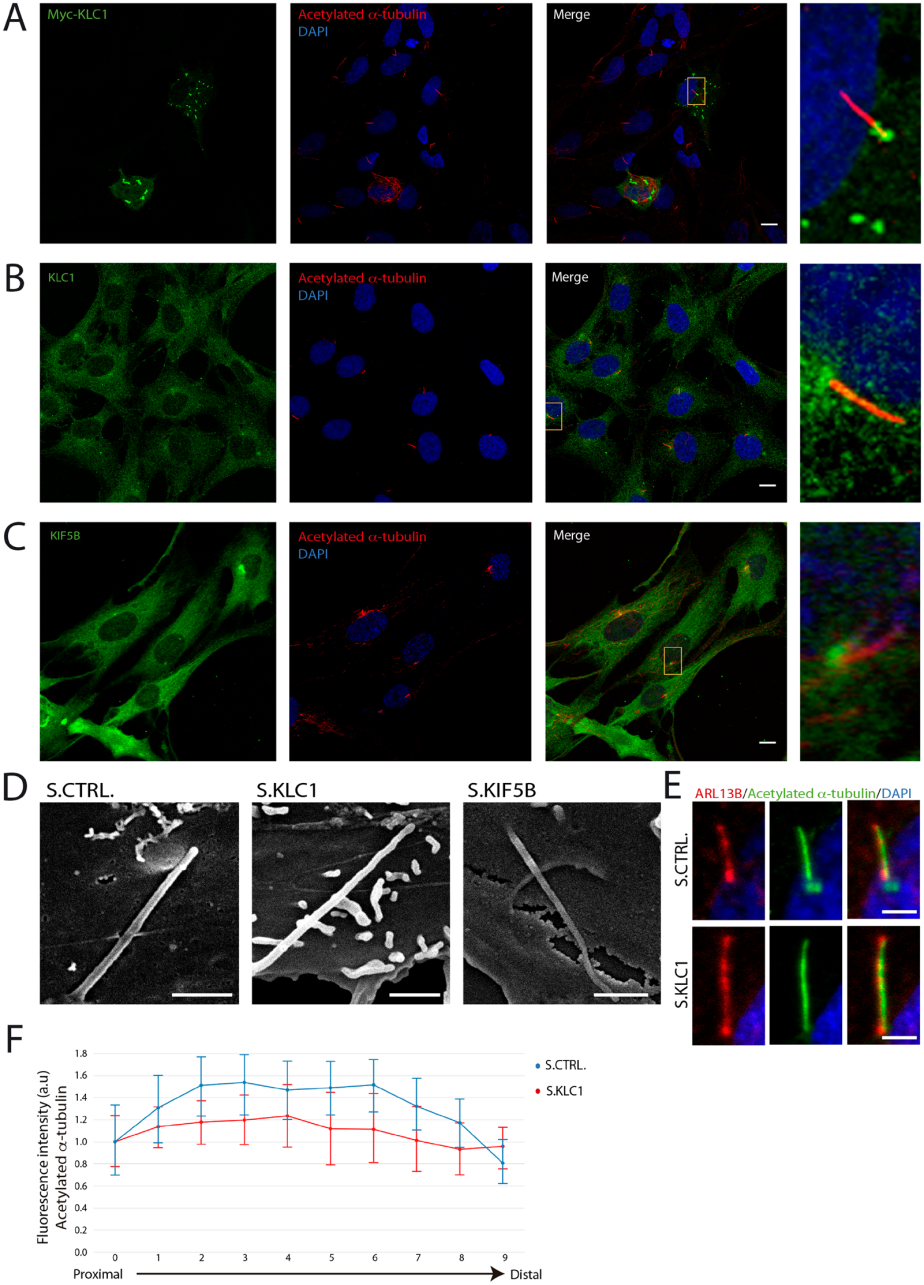
KLC1 and KIF5B are found at the base of cilia. Confocal microscopy analysis of hTERT-RPE cells. (**A**) Cells were transfected to overexpress Myc-tagged KLC1. In addition to cytoplasmic aggregates, a pool of Myc-KLC1 (anti-Myc, green) is found at the base of cilia (anti acetylated α-tubulin, red). (**B**) In addition to a diffuse cytoplasmic signal, the anti-KLC1 antibody shows an accumulation of endogenous protein at the base of cilia. (**C**) Similarly, endogenous KIF5B is found in the cytoplasm and also concentrated at the base of cilia. Yellow boxes mark the area that is magnified and showed in panels on the right. DAPI was used to stain nuclei. Scale bars correspond to 10 μm. (**D**) Scanning electron micrographs showing cilia in hTERT-RPE cells transfected with control (S.CTRL), KLC1 (S.KLC1) or KIF5B (S.KIF5B) stealth RNA oligos. Scale bars correspond to 1 μm. (**E**,**F**) The level of tubulin acetylation in control and KLC1 KD cilia was quantified measuring the fluorescence intensity of the signal obtained using the acetylated α-tubulin antibody (green). The anti-ARL13 (red) signal was used to mark the entire length of the cilium. Scale bars correspond to 2 μm. Each cilium was divided in 10 segments from base to tip (see methods) and the mean intensity was computed. For each experiment (KLC1 KD and control) a ten-point intensity profile was computed by averaging the measure of all cilia in each one of the regions of interest. These profiles are shown, normalized by the measure of its first region of interest. Vertical bars plot the 95% confidence interval about the mean.

As mentioned, different kinesins have been shown to localize at the base or inside the ciliary compartment and regulate cilia length by affecting axoneme maintenance through microtubule depolymerizing activities^17,28,29,33^. To gain insight on how kinesin 1 regulates cilia length we first evaluated the subcellular distribution of KLC1 and KIF5B in hTERT-RPE cells. We transfected cells with our Myc-KLC1 over-expressing construct and assessed the localization of the fusion protein using an anti-Myc antibody. Myc-KLC1 localized to cytoplasmic aggregates, one of which invariably localized at the base of the cilium as shown by the acetylated α-tubulin staining (Fig. 3A). These results are in agreement with a previous report showing KLC1 aggregates upon overexpression likely due to the interaction between heptad repeat domains in the protein^34^. Since overexpression can result in non-specific signal, we next used the anti-KLC1 antibody to evaluate the endogenous protein. We observed a wide cytoplasmic distribution and a signal at the base of cilia (Fig. 3B). Importantly, this localization pattern was no longer observed in KLC1 KD cells (Fig. S4A). Similarly, the anti-KIF5B antibody yielded a cytoplasmic diffuse staining and an accumulation of a specific signal at the ciliary base (Fig. 3C, Fig. S4B). The accumulation of signal at the ciliary base could be due to the high microtubule density present at the microtubule organizing center. In line with this, in none of the conditions tested we were able to detect KLC1 or KIF5B inside the ciliary compartment.

Another characteristic of elongated cilia in mutants of kinesins that regulate microtubule stability is that cilia present structural defects. For example *Kif7*^−/−^ mutant MEFs present long cilia that appear twisted and are unstable showing a reduction in the levels of acetylated tubulin, a post-translational modification associated with increased microtubule stability^28^. Unlike *Kif7*^−/−^ mutant cilia, our scanning electron microscopy analysis of elongated cilia in *KLC1* KD and *KIF5B* KD cells did not reveal overt structural defects (Fig. 3D). We also analyzed the fluorescence intensity of acetylated tubulin along the axoneme marking the entire length of cilia with an antibody against the ciliary protein ARL13B, as previously described^28^. While we cannot discard a subtle decrease in the level of acetylation in middle segments of KLC1 KD cilia, the pattern of tubulin acetylation was comparable to controls at both the proximal and distal end of cilia (Fig. 3E,F).

### Kinesin 1 and CCDC28B operate in a common pathway to regulate ciliogenesis

Our results therefore show that both CCDC28B and kinesin 1 physically interact and participate in a common cellular process, cilia length regulation, although with opposing roles. We next performed genetic interaction experiments to test whether these proteins act together in this process. We performed the analysis depleting KLC1 given that it yielded a more robust ciliary phenotype than targeting the KIF5s, likely due to functional overlap between heavy chains.

**Figure 4:**
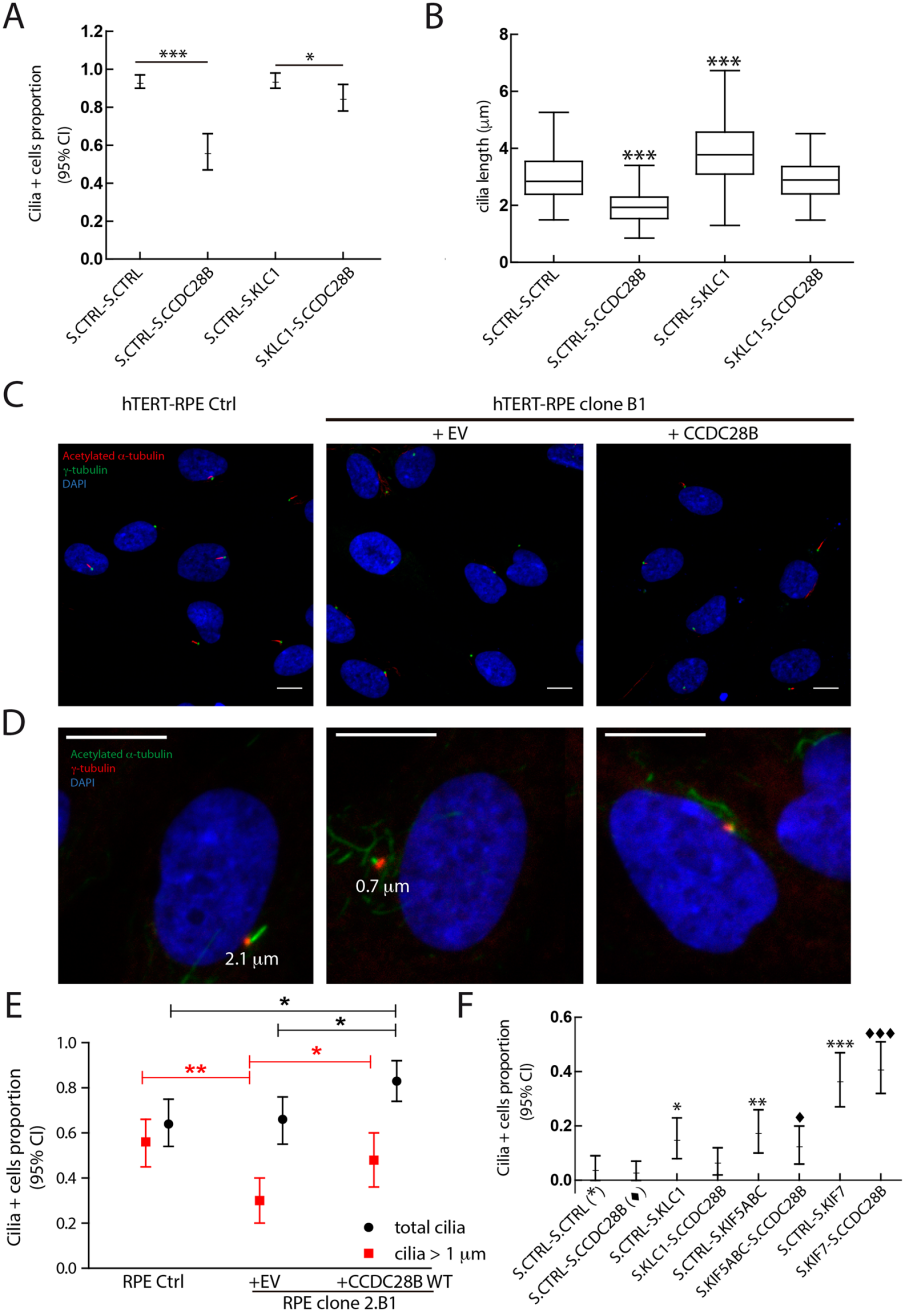
Kinesin 1 requires CCDC28B to regulate cilia length. (**A**) The proportion of cilia-positive cells is significantly reduced upon CCDC28B KD but not KLC1 KD (hypothesis test for proportions). Co-transfection of the KLC1 stealth oligo rescues the phenotype of CCDC28B KD cells. (**B**) CCDC28B KD (118 analyzed cilia) and KLC1 KD (155 cilia measured) cells show significantly shortened and elongated cilia respectively. Cilia were of control length in cells co-transfected with both stealth oligos (115 cilia). Statistical test: one-way ANOVA; ***P < 0.001. The amount of transfected oligos per condition was maintained constant using a control stealth oligo. (**C**) CCDC28B CRISPR clone B1 was analyzed by immunofluorescence and confocal microscopy using anti-acetylated tubulin (red), anti γ-tubulin (green) and DAPI (blue) to stain cilia, basal bodies and nuclei respectively. Untreated hTERT-RPE cells were used as control and CCDC28B CRISPR clone B1 transfected with pCS2+ _CCDC28B wt was used to show rescue and specificity of the cilia phenotype. (**D**,**E**) Clone B1 cells were analyzed to quantify the number of total cilia-positive cells (acetylated α-tubulin signal irrespective of length) and cells bearing “long” cilia of at least 1 μm. Scale bars represent 10 μm. (**E**) Quantification shows that while the total number of cilia is similar between hTERT-RPE controls and Clone B1, the later presents significantly less cilia of at least 1 μm. Importantly, the proportion of cells bearing 1 μm cilia is rescued upon transfecting wt CCDC28B. 80 cells were analyzed for control and clone B1 cells, and 70 cells were scored for clone B1 overexpressing CCDC28B. Statistical test used: hypothesis test for proportions. Data are representative of three independent experiments. (**F**) Genetic interaction experiment using CCDC28B CRISPR clone B1. KLC1 KD and KIF5ABC KD result in a mild rescue (compared to that of KIF7) that is abolished or diminished by further CCDC28B KD for KLC1 and KIF5s respectively. KIF7 KD rescues the ciliary phenotype irrespective of the presence of CCDC28B. The experiment shown is representative of four independent experiments. The total number of cells scored for this experiment is 89 Ctrl.-Ctrl., 96 Ctrl.-CCDC28B, 91 Ctrl.-KLC1, 86 KLC1.-CCDC28B, 84 Ctrl.-KIF5ABC, 86 KIF5ABC-CCDC28B, 84 Ctrl.-KIF7, 104 KIF7.-CCDC28B. Statistical test: hypothesis test for proportions. *Indicate comparison to Clone B1 transfected with control oligos (S.CTRL-S.CTRL). ^♦^Indicate comparison to Clone B1 transfected with control oligo and CCDC28B oligo (S.CTRL-S.CCDC28B). ^*/♦^*P* < 0.05; ^**^P < 0.01; ^***/♦♦♦^*P* < 0.001. Scale bars correspond to 10 μm.

Upon targeting both CCDC28B and KLC1 we expected that the final phenotype would correspond to that of the protein lying downstream in a common pathway. We transfected hTERT-RPE cells with our RNA oligos targeting *CCDC28B* and *KLC1*, either alone or in combination, maintaining the total amount of transfected RNA constant with control oligos, and measured the proportion of cilia-positive cells and cilia length. The proportion of cilia-positive cells was reduced only in CCDC28B KD cells, a phenotype that was partially rescued by KLC1 KD (Fig. 4A). Of note, knockdown of CCDC28B results in shortened cilia and therefore, a reduction in the proportion of cilia-positive cells does not necessarily indicate a ciliogenesis defect as cells bearing extremely short cilia would not be scored as positive (for example see Fig. 4E and text below). Regarding cilia length, cells transfected only with CCDC28B or KLC1 RNAi oligos presented shorter and longer cilia respectively as expected (CCDC28B KD: 1.9 μm; KLC1 KD: 3.8 μm; control cells: 2.9 μm; Fig. 4B). When co-transfected, the two effects were compensated and cilia length was comparable to controls (2.9 μm; Fig. 4B). Therefore, we could not distinguish between a *bona fide* interaction from an additive effect of perturbing two independent cilia regulatory pathways. Since this result could be due to only partially depleting CCDC28B and KLC1, we decided to perform this study in a null background for one of the genes.

Given the broad role of kinesin 1 in intracellular transport we first favor the possibility of CCDC28B being downstream in a putative common pathway. Additionally, by targeting *CCDC28B* we expected to minimize the chances of observing pleiotropic effects associated with kinesin 1-impairment that could hinder the analysis. We generated five different lentiviral vectors encoding a guide RNA (gRNA), targeting either exon 2 (first coding exon) or exon 3 of *CCDC28B*, and the Cas9 nuclease^35,36^ and transduced hTERT-RPE cells. We selected transduced cells with puromycin and assessed levels of CCDC28B 3 and 10 days after transduction. While we did observe a reduction in CCDC28B levels at day 10 for most gRNAs, in some cases there appeared to be a recovery of CCDC28B when compared to the knockdown at day 3 (Fig. S5A, see gRNAs #3). We therefore decided to establish CRISPR CCDC28B clones (using gRNA #1, exon 2) by cell sorting and single cell deposition in 96-well plate.

A large number of clones stop dividing at early passages and thus a cell line could not be established. We were able to obtain DNA from one of these wells and upon sequencing determined that it carried two frameshift (fs) mutations leading to premature stop codons (PTCs) due to insertions of 2 and 1 bases respectively (not shown). In the ones that did grow, we checked CCDC28B levels by western blot and unexpectedly detected the protein in all cases (Fig. S5B). Altogether these results suggested that complete absence of CCDC28B could be inhibiting cell proliferation in hTERT-RPE cells. Despite this, the clones that were analyzed presented a marked reduction in cilia length and the proportion of cilia-positive cells (representative results are shown in Fig. 4C–E). We determined the genomic mutations in some of these clones and found that three presented one allele with a 1 or 2 bp insertion leading to frameshifts and PTCs. The other alleles that we detected were predicted to encode *CCDC28B*, albeit with loss/gain of few residues with the exception of clone B1.

Clone B1 presented a 2 bp insertion in one allele and a 220 bp deletion in the second allele, eliminating the last 93 nucleotides of exon 2 (31 residues, from R25 to R55) and the first 127 bases of intron 2 (c.72_164 + 127Del; Fig. S6). However, despite this genotype we detected CCDC28B of seemingly wild type molecular weight and at comparable levels to controls by western blot (Fig. S5B) and not 50% of a lower molecular weight CCDC28B as expected. We analyzed cDNA by sequencing and semi-quantitative RT-PCR. We determined that the deletion triggered the utilization of a cryptic splice donor site 60 bp into the remaining intron 2. This aberrantly spliced mRNA was therefore expected to encode a protein that loses 31 wild type residues but that incorporates 20 novel residues from retained intron 2 sequences before continuing in frame with exon 3 (Fig. S6). The predicted molecular weight of this mutant protein differs by 1 KDa with the wild type protein. By semi-quantitative RT-PCR we detected only the aberrantly spliced *CCDC28B* mRNA and at lower levels than the wild type mRNA from control cells (Fig. S5C). Thus, the mRNA encoded by the allele carrying the fs mutation that leads to a PTC in exon 3 (Fig. S6), was not detected. This result is in agreement with our previously reported data showing that an allele carrying a PTC in exon 3 was degraded by nonsense mediated decay^10^. However, these results suggest the possibility of a post-transcriptional compensatory mechanism to maintain CCDC28B levels in clone B1 that will need further evaluation.

We selected B1 to continue as it was likely the clone with the most compromised CCDC28B function since it expressed only a mutated protein missing 31 amino acids of the wild type sequence. CCDC28B B1 presented reduced ciliation with the scarce cilia present being too short to assess length (Fig. 4C). Therefore, in this analysis we only quantified the proportion of cilia-positive cells. As stated before, given the level of resolution of our microscopy, this parameter is not an exact measure of ciliogenesis, particularly in cases where cilia are extremely short as clone B1. Therefore, we carried out a more detailed analysis scoring both cells with a cilium of 1 μm or more and the total number of cilia (all cells with a visible acetylated tubulin mark irrespective of size and shape) (Fig. 4D). Interestingly, while the proportion of total cilia was comparable between clone B1 and control (Fig. 4E, black data), the proportion of “longer” cilia was significantly reduced in clone B1, a defect that was rescued by overexpressing CCDC28B: the proportion of cilia positive cells (1 μm or more) was 0.56 in control hTERT-RPE cells, was reduced to 0.30 in the B1 clone and was 0.48 upon rescue (Fig. 4E, red values). Overexpression of CCDC28B in clone B1 did result in an increase in the proportion of total cilia and thus we cannot discard that CCDC28B levels may also impact ciliogenesis (Fig. 4E). Therefore, our data indicates that the main defect in CCDC28B clone B1 is cilia shortening.

Knocking down KLC1 or KIF5 (all three heavy chains), which normally elongates cilia, resulted in a subtle rescue in the proportion of cilia-positive cells in CCDC28B clone B1 (Fig. 4F). Since this effect could rely on the remaining CCDC28B activity present in this clone we further targeted CCDC28B by RNAi. Importantly, knocking down the remaining CCDC28B by RNAi completely abrogated the KLC1 KD mediated rescue, and to a lesser extent that of KIF5s (Fig. 4F). In contrast, targeting KIF7, which normally restrains cilia elongation by promoting microtubule depolymerization inside the cilium^28^ and therefore likely acts in a CCDC28B-independent manner, resulted in a significant and larger rescue in the proportion of cilia-positive cells compared to kinesin 1, and was independent of the CCDC28B RNAi treatment (Fig. 4F). Thus, our results indicate that kinesin 1 requires CCDC28B to regulate cilia length.

### Kinesin 1 regulates the subcellular distribution of CCDC28B

**Figure 5:**
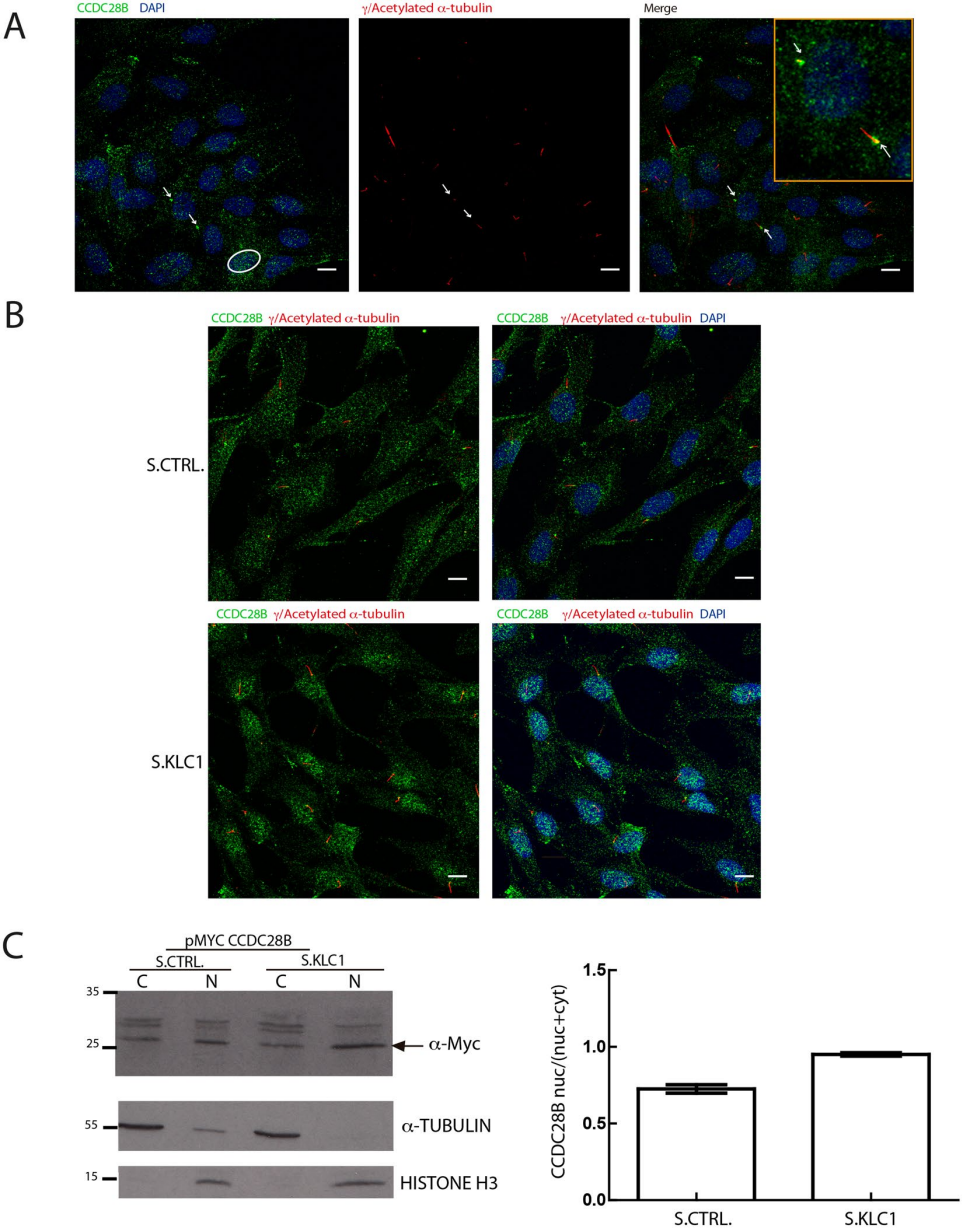
KLC1 plays a role regulating the sub-cellular distribution of CCDC28B. (**A**) Confocal microscopy analysis of endogenous CCDC28B (green) in hTERT-RPE cells showing localization of the protein in the cytoplasm, pericentriolar region/basal body (arrows illustrate examples in a ciliated and a non-ciliated cell, higher magnification in yellow box) and the nucleus (circle). Basal bodies and cilia axoneme were stained with anti-γ- and anti-acetylated α-tubulin respectively (red). (**B**) CCDC28B (green) signal is increased in the nucleus upon KLC1 KD (S.KLC1; lower panels) compared to control cells (S.CTRL; upper panels). In both (**A**,**B**), DAPI was used to stain nuclei. Scale bars correspond to 10 μm. (**C**) Sub-cellular fractionation assay using hTERT-RPE cells transfected with a Myc-CCDC28B expressing plasmid together with stealth control (S.CTRL) or stealth KLC1 (S.KLC1). CCDC28B is present in both the cytosolic and nuclear fractions in control cells and accumulates in the nuclear fraction in KLC1 KD cells. The membrane was cut at the 35 KDa ladder band. The blot incubated with the α-Myc to visualize CCDC28B was stripped and probed with α-Histone. The graph shows the nuclear/total (nuclear + cytoplasmic) ratio obtained by quantifying the western blot bands by densitometry. α-tubulin was used to normalize the nuclear intensity of CCDC28B and compensate for the cytosolic contamination in the nuclear fraction.

Our previous results suggest that kinesin 1 could regulate the activity of CCDC28B in cilia length regulation. Being a microtubule associated molecular motor we hypothesized that it could do so by affecting the subcellular distribution of CCDC28B. Using our rabbit polyclonal anti-CCDC28B antibody^13^ we observed signal at the pericentriolar region at the base of cilia, as was previously reported^10^, but also in a diffuse cytoplasmic pattern and in the nuclear compartment (Fig. 5A). Next, we evaluated the localization of CCDC28B in KLC1 KD cells. We did not observe overt changes in the pericentriolar localization of CCDC28B. Variable among cells, we observed an unexpected increase in the nuclear stain of CCDC28B upon depletion of KLC1 (Fig. 5B). Depleting the KIF5s yielded comparable results (Fig. S7). Given the observed variability in our immunocytochemistry assays we quantified this finding performing subcellular fractionation and western blot comparing the nuclear/total ratio of CCDC28B in control and KLC1 KD cells. KLC1 KD resulted in an increase in nuclear CCDC28B (Fig. 5C). Thus, our results suggest that kinesin1 could be involved in regulating CCDC28B subcellular distribution.

### Deletion of a predicted NLS impairs function of zebrafish ccdc28b

To follow up on the previous finding we decided to evaluate whether a predicted nuclear localization signal (NLS), encompassing residues 4 to 10 in CCDC28B (http://www.moseslab.csb.utoronto.ca/NLStradamus/http://elm.eu.org; Fig. S8A)^14,37,38^, is functional. This NLS is conserved between human and zebrafish and therefore we deleted residues 4 to 10 (Δ4–10) in both orthologs to generate NLS mutants. We determine that the human CCDC28B NLS mutant retains its capacity to interact with KLC1 (Fig. S8B). We attempted to establish whether the NLS deletion affected the localization of CCDC28B by immunofluorescence but the protein produced at high levels upon transfection did not recapitulate the endogenous localization pattern resulting in a non-informative, largely homogeneous signal (not shown), probably due to the small size of CCDC28B (approximately 25 KDa). We then transfected cells with human wild type (wt) and NLS mutant CCDC28B and performed cell fractionation followed by western blot. Upon overexpression of CCDC28B we observed both wt and NLS mutant protein in the cytosolic and nuclear compartments, a finding that is in agreement with our immunocytochemistry results. However, the nuclear levels of NLS mutant CCDC28B were reduced compared to wt suggesting that the NLS motif could be functional (Fig. S8C). Similarly, the zebrafish ccdc28b NLS mutant expressed in hTERT-RPE cells also showed reduced nuclear entry (Fig. S8D).

We then attempted to test the functional relevance of this CCDC28B NLS motif in rescue experiments. When transfecting human CCDC28B wt and NLS mutant we repeatedly observed that the levels of the later were lower than the wild type protein (for an example see Fig. S8B, lysates). Therefore, we could not use it to rescue the B1 clone as a reduced rescue could be due to impair function or simply to lower levels than wt. In contrast, the zebrafish wild type and NLS mutant ccdc28b were expressed at comparable levels when we injected mRNA in embryos (Fig. S8E). We therefore decided to use zebrafish to evaluate the functionality of this conserved NLS motif.

**Figure 6:**
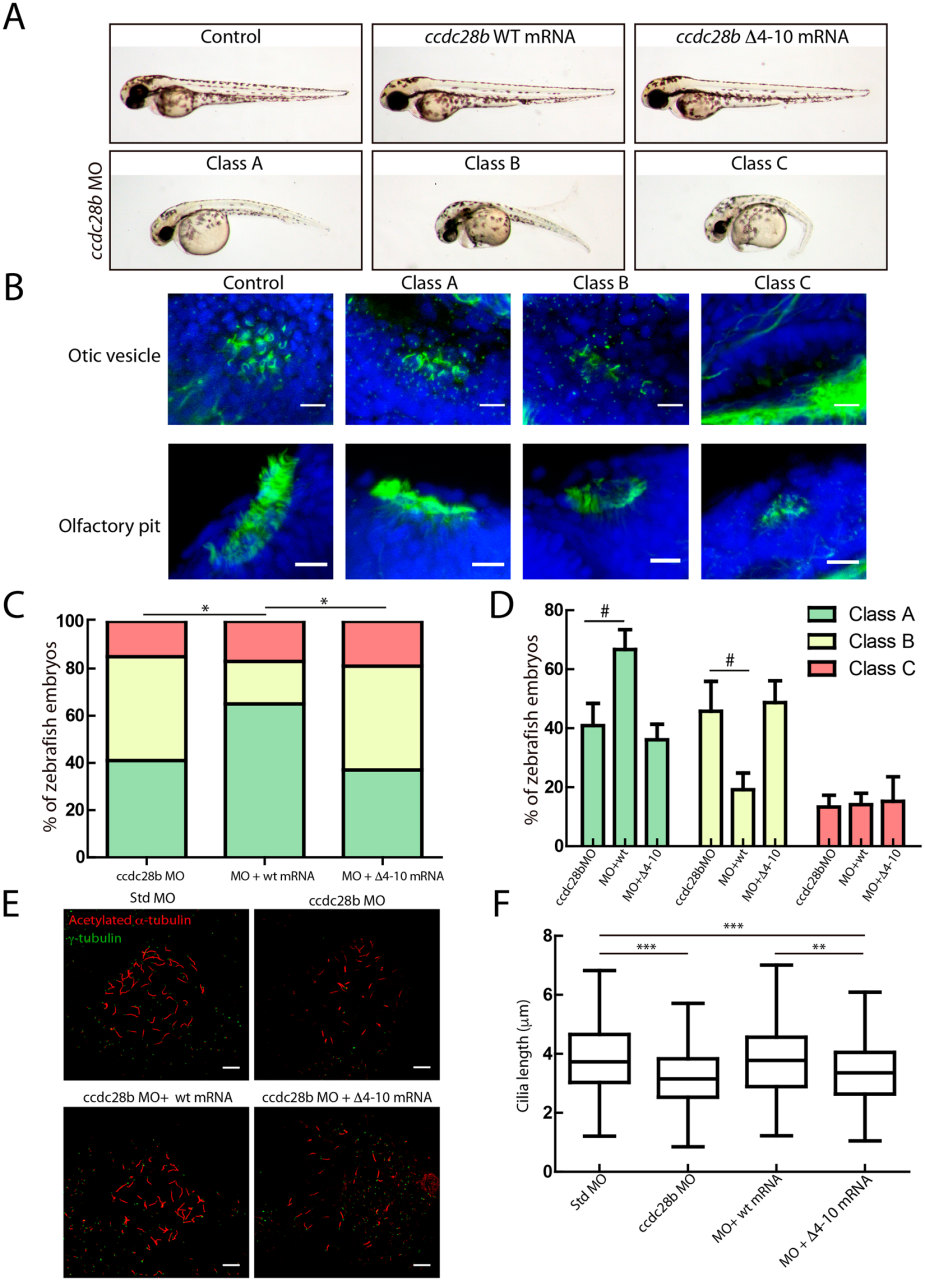
A Δ4–10 *ccdc28b* mutant does not rescue the morphant phenotype in zebrafish. (**A**) 48 hpf control and injected zebrafish embryos are shown. While injecting either wt or mutant *ccdc28b* mRNA does not result in phenotypic alterations, injection of the ccdc28b MO results in a range of phenotypes from mild to severe corresponding to Class A to C respectively. Representative images of each class are shown. (**B**) The severity of the external morphological phenotype in the different classes correlates with an increasing perturbation of ciliated tissues. Otic vesicle and nasal pit are shown. (**C**) The class distribution upon injection of morpholino alone (*ccdc28b* MO) and morpholino co-injected with either wt or Δ4–10 mutant *ccdc28b* mRNA was compared. Data from five independent injections were pooled reaching 128 *ccdc28b* MO, 65 *ccdc28b* MO + wt mRNA and 63 *ccdc28b* MO + Δ4–10 *ccdc28b* mRNA embryos. A rescue of the phenotype was observed only upon injecting the wt mRNA but not the Δ4–10 mutant. Statistical test: χ^2^; *P < 0.01. (**D**) The percentage of embryos in each phenotypic class was calculated for the five individual experiments. The mean and SEM (bars) are plotted. The differences between conditions (#) were analyzed using the Wilcoxon rank test (*P* = 0.06 for class A; *P* = 0,05 for class B) and paired t-test assuming normal distribution (*P* = 0.02 for both class A and class B). (**E**) Cilia and basal bodies in KV were visualized with anti-acetylated (red) and anti-γ-tubulin (green) respectively. Representative images for control, *ccdc28b* MO, and *ccdc28b* MO embryos co-injected with either *ccdc28b* wt or Δ4–10 mRNA. (**F**) Cilia length was measured using ImageJ (355 cilia for Std. MO, 336 for *ccdc28b* MO, 276 for *ccdc28b* MO + wt mRNA, 266 *ccdc28b* MO + Δ4–10 mRNA). Cilia are significantly shorter in *ccdc28b* MO embryos and length is rescued upon co-injection of wt ccdc28b mRNA but not with the Δ4–10 ccdc28b mRNA. Statistical test: Kruskal Wallis. ^**^P < 0.01, ^***^P < 0.001. Scale bars correspond to 10 μm.

Knockdown of *ccdc28b* in zebrafish with morpholinos (MO; Fig. S8F) results in cilia defects in different tissues and embryos characterized by a significant shortening of the body axis, increased body curvature, reduced eye size, craniofacial alterations and defects in pigmentation (Fig. 6). These phenotypes are characteristic of ciliary mutants and importantly, we have shown previously that are specific of *ccdc28b* since they can be rescued by the co-injection of wild type (wt) zebrafish *ccdc28b* mRNA^14^. To further confirm their specificity however, we performed genome editing in zebrafish embryos targeting exon 2 of *ccdc28b* by CRISPR/Cas9. We designed and tested four different gRNAs (Fig. S9A and data not shown) both alone and in combination and assessed phenotypes at 48 hpf in the F0. Injection of individual gRNAs did not produce major phenotypic changes. Injecting a combination of two gRNAs however resulted in a range of phenotypes that as expected, were highly variable in penetrance in the F0. Importantly however, the different phenotypes that we observed were comparable to the phenotypes of our morphants thus further validating the specificity of our MO-generated phenotypes (Fig. S9B).

We next tested whether Δ4–10 (NLS) mutant ccdc28b could rescue the phenotype of *ccdc28b* morphant embryos. We co-injected 2–4 cell zebrafish embryos with our validated *ccdc28b* splice-blocking MO either alone or in combination with wt or Δ4–10 mutant *ccdc28b* mRNA. A control MO and the individual mRNAs were injected as controls. For each condition, two independent investigators (one blinded to the experiment) classified morphant embryos into three different categories according to their external phenotype (Fig. 6A): class A ranged from embryos characterized by a mild shortening of the body with a slight curvature to wild type looking embryos; class B presented a pronounced shortening of the body axis, ventral curvature, pigmentation and craniofacial defects; class C were characterized by a severe shortened and curved body, and pronounced pigmentation and craniofacial defects. By whole-mount immunofluorescence of embryos stained for acetylated α-tubulin we observed that the severity classes correlated with the degree of defects in ciliated tissues, assessing olfactory pit and otic vesicle (Fig. 6B), similarly to what we observed with our CRISPR mutants (Fig. S9B).

To assess rescue we scored embryonic phenotypic classes in five independent experiments. For each one we injected embryos obtained from one female fish to generate all the experimental conditions (MO alone, MO + wt mRNA and MO + Δ4–10 mRNA), and all the controls needed for validation (non-injected, wt mRNA alone and Δ4–10 mRNA alone). By doing this we eliminated the variability that could be introduced by using different batches of embryos but resulted in low number of embryos per condition in each individual injection.

Therefore, we first analyzed the individual experiments (Fig. S10) and then proceeded to pool all the data and compare the overall distribution of phenotypic classes between conditions. The analysis showed that the distribution of classes in the MO + wt mRNA was significantly different than the one observed in the MO alone or the MO + Δ4–10 mRNA. The *ccdc28b* MO alone resulted in 41% class A, 46% class B and 13% class C. Co-injecting the wt *ccdc28b* mRNA increased the percentage of mildly affected class A embryos (67%) and reduced the more severe Class B to 19% (χ^2^ test, *P* = 0.005; Fig. 6C). In contrast, co-injecting the Δ4–10 *ccdc28b* mRNA did not rescue the morphant phenotype resulting in a distribution comparable to the MO alone with 36% class A, 49% class B and 15% class C (χ^2^ test, *P* = 0.87; Fig. 6C), while it was significantly different than the wt rescue (χ^2^ test, *P* = 0.007; Fig. 6C). To analyze this effect further we compared the mean percentage of embryos for each class in each experimental condition (Fig. 6D). This analysis confirmed that the difference between MO alone and MO + wt mRNA is caused by an increase in class A and a reduction of class B in the rescue condition (Wilcoxon rank test, *P* = 0.06 and *P* = 0.05 respectively; assuming normal distribution, paired t-test, *P* = 0.02 in both cases).

In addition to the external phenotype of embryos we tested whether the Δ4–10 *ccdc28b* could rescue cilia defects specifically. We processed age-matched 12-somite embryos to visualize cilia in the Kuppfer’s vesicle (KV) by confocal microscopy using antibodies against acetylated- and γ-tubulin (Fig. 6D). We measured in excess of 250 cilia per condition comparing controls (standard MO), *ccdc28b* MO, and *ccdc28b* MO co-injected with either wt or Δ4–10 *ccdc28b* mRNA. As expected, the *ccdc28b* MO resulted in significantly shortened cilia with a median of 3.1 μm compared to 3.7 μm in controls (*P* = 4.10E-13). Co-injecting the wt mRNA rescue the phenotype of the morphants restoring cilia length to control values with a median of 3.8 μm (*P* = 0.339). In contrast however, the cilia length of morphant embryos co-injected with *ccdc28b* Δ4–10 was significantly different from controls (a median of 3.4 μm; *P* = 4.18E-05) and embryos co-injected with wt *ccdc28b* mRNA (*P* = 0.0051) (Fig. 6E). These results indicate that residues 4 to 10, encompassing a NLS, are important to maintain function of zebrafish ccdc28b.

## Discussion

CCDC28B is a 200 amino acid protein characterized primarily by a coiled coil domain located at its C-terminal region, a motif involved in protein-protein interactions^14^. Therefore, we have hypothesized that the main role of this BBS associated protein is to mediate the function of proteins that physically interact with it, for example the BBS proteins and SIN1^10,13^. In this context we reasoned that it was vital to continue with the identification of proteins interacting with CCDC28B, as this would represent a critical step to understand its cellular function. Here we uncovered a link between CCDC28B and kinesin 1, providing novel insights to understand the function of CCDC28B and importantly, linking a major kinesin motor with cilia length regulation.

As mentioned, kinesin 1 is composed of both heavy and light chains. There are three different KIF5 heavy chains that can be part of the kinesin 1 molecular motor: KIF5A, KIF5B and KIF5C. By knocking down each one individually or in combination, our data show that KIF5B is the main heavy chain involved in this novel cilia-related function in hTERT-RPE cells. In contrast, we found that KIF5A is not heavily expressed in this cell line while KIF5C KD alone was not sufficient to affect cilia. However, KIF5C KD in combination with KIF5B KD consistently resulted in a mild increase in ciliary length compared to KIF5B KD alone, although not statistically significant. Thus, KIF5C could play at least a minor role regulating cilia. It will be interesting to test whether the contribution of different kinesin heavy chains to ciliogenesis varies between different cell types.

Different kinesins have been shown to participate in ciliogenesis. For example kinesin 13 (KIF24, KIF2A), kinesin 4 (KIF7), and kinesin 8 (KIF19A) affect cilia by modulating microtubule dynamics at either the basal body, the ciliary axoneme or in the cytoplasm^26–33^. Although it is difficult to conclude based on negative findings, a role of kinesin 1 modulating axonemal microtubules is unlikely since we were not able to detect KLC1 or KIF5B inside cilia. Supporting this notion, KLC1 KD or KIF5B KD did not produce gross changes in cilia ultrastructure or axonemal tubulin acetylation levels, features that have been reported upon KIF7 KD, a kinesin that enters the cilium and has microtubule depolymerizing activity^28^. We did detect KLC1 and KIF5B at the pericentriolar region at the base of cilia, which could be due to the increased density of microtubules in the region of the microtubule organizing center. At this location, microtubule depolymerization by KIF2A has been shown to promote cilia disassembly^27^. However, kinesin 1 has not been reported to present a microtubule depolymerizing activity but rather to stimulate microtubule elongation in the cytoplasm through the modulation of the c-Jun N-terminal kinase (JNK) pathway^39^. Furthermore, our biochemical and genetic studies underscore a different role for kinesin 1 on cilia linked to CCDC28B.

We show that CCDC28B can physically interact with the kinesin 1 motor and our genetic interaction studies strongly suggest that kinesin 1 and CCDC28B regulate cilia length through a common pathway. Using our hTERT-RPE CRISPR CCDC28B cell line we were able to show that KLC1 KD or KIF5B KD require CCDC28B to regulate cilia length. In contrast, KIF7 KD, which is known to affect axonemal microtubule dynamics, resulted in cilia elongation and rescue independently of CCDC28B. Of note, we were unable to isolate a complete CCDC28B knockout cell line suggesting that at least a minimal activity of this protein is required for hTERT-RPE cells to grow. Furthermore, clone B1, albeit carrying only one CCDC28B coding allele presented levels of CCDC28B comparable to controls. This may indicate the presence of compensatory mechanisms maintaining CCDC28B levels. Our RT-PCR data suggest that if this is the case it would be a post-transcriptional event for example increasing mRNA occupancy and translational rates or modulating protein turnover. Further studies will require to test these possibilities.

Therefore, we report here a previously unknown role for kinesin 1 in cilia length regulation, an activity that it achieves, at least in part, through the Bardet-Biedl associated protein CCDC28B. In addition, finding the link between CCDC28B and a microtubule based motor such as kinesin 1 provided a new entry-point to continue dissecting the still largely unknown mechanism by which CCDC28B regulates cilia length. Altogether, our data support a model where the function of kinesin 1 is to inhibit the pro-ciliogenic role of CCDC28B, which lies downstream in the pathway. Given the known function of kinesin 1, we reasoned that it could affect the function of CCDC28B by modulating its subcellular distribution. Interestingly, our results suggest this could be the case since depletion of KLC1 and KIF5B resulted in changes in CCDC28B localization: we observed nuclear accumulation of CCDC28B upon kinesin 1 KD, although other changes in localization cannot be discarded. Nuclear entry requires directing the target protein towards the nuclear envelope, often depending on the microtubule network and the tight association between the pericentriolar region/centrosome and the nucleus. Thus, kinesin 1, a microtubule plus end molecular motor, could be transporting CCDC28B away from the pericentriolar region and nucleus. In ciliated cells, kinesin light chains were shown to interact with rootletin, the main component of the ciliary rootlet, a structure that has been proposed to function as a site for loading cargo onto kinesin 1 motors^34^. In this context, KLC1 could be in charge of loading CCDC28B onto KIF5B-based motors at the pericentriolar region. This type of effect has been reported for other kinesin 1 cargoes such as disrupted in schizophrenia (DISC1), a centrosomal protein that requires kinesin 1 to translocate away from the pericentriolar region and into the axon in neurons^40–42^.

Driven by the observed changes in cellular localization we analyzed CCDC28B further and show that both human and zebrafish CCDC28B likely possess a functional NLS domain at their N-ter. Moreover, deleting this motif in the zebrafish ortholog resulted in a protein that behaves as a null in our *in vivo* rescue assays. Eliminating amino acids 4–10 comprising the NLS could result in a null ccdc28b because it impairs nuclear translocation of the protein but also by other mechanisms that we cannot discard at this point. For example, it could impact protein folding and hence function although, at least for human CCDC28B, the fact that the NLS mutant protein retains its ability to interact with KLC1 suggests that is not the case. Also, deletion of residues 4–10 could impact the ability of CCDC28B to localize to the base of cilia, a possibility that we could not assess due to difficulties assessing localization upon overexpression of the mutant protein. Finally, the deletion could affect a yet unknown functional motif in the protein that is relevant to cilia length regulation. Further studies will be required to fully dissect the contribution of this NLS motif.

Our results therefore raised the intriguing possibility of CCDC28B playing a cilia-associated role in the nuclear compartment. Thus, one relevant question is whether the CCDC28B interactors, BBS proteins or SIN1, could also play a relevant function in the nucleus. Interestingly, we have shown that BBS7, which interacts with CCDC28B^10^, enters the nucleus and modulates gene transcription through an interaction with RNF2, a member of the polycomb chromatin remodeling complex^43^. This finding was reinforced recently by a report showing that BBS6 actively translocates between the cytoplasm and nucleus and interacts with the SWI/SNF chromatin remodeling protein SMARCC1 thus affecting gene transcription^44^. In the case of SIN1, the other reported CCDC28B interacting protein linked to cilia length regulation^13^, there are at least five isoforms produced by alternative splicing that present a dynamic sub-cellular localization being found in the plasma membrane, cytoplasm, nucleus, and in the case of the shorter SIN1γ, the basal body^45–47^. Thus, it is tempting to speculate that CCDC28B, together with kinesin 1, could play a role modulating the subcellular localization and function of BBS proteins or SIN1.

In addition to nuclear enrichment of CCDC28B we could have expected an accumulation of CCDC28B in the pericentriolar region upon depletion of KLC1/KIF5B. However, this was not evident although this could be expected if CCDC28B rapidly translocates into the nucleus. Therefore, it is tempting to speculate that kinesin 1 could also regulate CCDC28B levels in the pericentriolar region. CCDC28B interacts with BBS4, a protein mainly localized at the pericentriolar region^48^. Interestingly, it has been postulated that modulating the levels of BBS4 at the pericentriolar region could represent a major regulatory point for the formation of the BBSome, a complex of BBS proteins with a critical role in cilia formation, maintenance and function^49–51^.

Future research will have to address the question of whether CCDC28B, via kinesin 1, can in fact regulate the subcellular distribution and function of its interactors, both in the nucleus and in the cytoplasm, and whether this could impact their function, in particular, regarding ciliogenesis. Fully determining the function of CCDC28B will allow us to understand its role in the pathogenesis of BBS thus providing insight to fully dissect the cellular and molecular basis of the syndrome.

## Methods

### Yeast two-hybrid screen

We performed the Cytotrap yeast two-hybrid screen following the manufacturer’s instructions (Stratagene) using CCDC28B (NM_024296) cloned into the pSOS bait vector and a human fetal brain library as prey (Stratagene).

### Cell culture and transfections

We maintained Hek293 cells in Dulbecco’s modified Eagle medium (DMEM; Invitrogen) supplemented with 10% fetal bovine serum at 37 °C in 5% CO_2_ and hTERT-RPE cells in a 1:1 mix of Dulbecco’s modified Eagle medium (DMEM) and F12 (Invitrogen) supplemented with 10% fetal bovine serum and 0.01 mg/ml hygromicin B, at 37 °C in 5% CO_2_. We transfected cells using either FuGene (Promega) for plasmids or LipofectamineRNAiMax for stealth double stranded RNA oligos following the manufacturers recommendations (Invitrogen). Control cells were transfected with the recommended Stealth RNAi Low GC Negative Control duplex (Invitrogen).

### VHH against CCDC28B

We immunized a llama with recombinant CCDC28B and prepared a VHH phage display library from peripheral blood leukocytes. We selected the VHH to CCDC28B by panning on the polystyrene-adsorbed antigen and confirmed its reactivity by ELISA and western-blot. For immunodetection of CCDC28B and pull-down experiments, we produced the *in vivo* biotinylated VHH as described^52^. All activities were performed in accordance with international guidelines with special care to establish high standards of biosafety and animal welfare. All protocols were authorized by the ethics committee for animal experimentation of the Universidad de la República (UdelaR), Montevideo, Uruguay, and the animal ethics committee of the Lecocq Municipal Zoo of Montevideo, Uruguay.

### Co-immunoprecipitations

We transfected Hek 293 cells in 10 cm dishes and harvested them 48 hours post-transfection to obtain cell lysates using CoIP buffer (50 mM Tris-HCl pH 7.5, 150 mM NaCl, 1% NP-40) supplemented with a protease inhibitor cocktail (Sigma) and 100 mM Na_3_VO_4_. The lysates were cleared by centrifugation at 17000 g for 15 minutes at 4 °C. The supernatants were incubated with the appropriate antibody immobilized onto protein A/G Sepharose beads (Invitrogen) or with biotinylated-VHH against CCDC28B immobilized onto Streptavidin-Agarose resin (Pierce), overnight at 4 °C under rotation. After extensive washing of the beads, proteins were recovered with Laemmli’s sample buffer.

### Sub-cellular fractionation and Western blot

We prepared nuclear and cytoplasmic fractions from hTERT-RPE cells using a hypotonic buffer (100 mM Hepes pH7.9, 1 mM EDTA, 0.1 mM EGTA) to swell cells prior to homogenization and addition of buffer T (3% Triton X-100 and 10 mM Hepes pH 7.9). We then separated intact nuclei and the cytoplasmic fraction by centrifugation at 800 g for 5 minutes. RIPA buffer (50 mM Tris pH 7.4, 150 mM NaCl, 5 mM EDTA, 1% Na-deoxycholate and 1% NP-40) was used to solubilize proteins of the nucleoplasmic fraction. We performed western blots with the corresponding primary antibodies: anti-KIF5B and anti-KLC1 from Abcam, anti-Histone H3 from Cell Signaling, anti-HA and anti-Myc from Sigma, and rabbit polyclonal anti-CCDC28B custom made by Genscript using a human peptide. We quantified western blot bands by densitometry using the ImageJ software (Image Processing and Analysis in Java, NIH).

### Immunofluorescence, confocal microscopy and image processing

We cultured hTERT-RPE cells on glass coverslips. 24 hours post-transfection with Stealth RNAi we serum starved cells for 48 hours in order to induce ciliation. We fixed cells with cold methanol at −20 °C or 4% PFA at 4 °C for 10 minutes, permeabilized for 10 minutes with 0.1% Triton X100 and blocked with 5.5% FBS for 1 hour. We incubated with the appropriate primary antibodies for 1–2 hours at room temperature and used secondary antibodies coupled to either Tetramethylrhodamine or Alexa Fluor 488 (Invitrogen). We stained basal bodies and cilia (in cells and zebrafish) using anti-γ-tubulin and anti-acetylated α-tubulin antibodies (Sigma) respectively and isotype specific secondary antibodies (Invitrogen). ARL13B was detected using a specific antibody from Abcam. 4,6-diamidino-2-phenylindole (DAPI) was used to stain DNA. Images were obtained in a Leica TCS-SP5 confocal microscope using a 63x oil 1.4 NA objective.

To evaluate levels of acetylated tubulin Z-stack images of individual cilia were semi-automatically processed in the image processing package FIJI^53^ and data was analyzed using Pyhton scripts. For each cilium, the 3D distal and proximal points were manually defined by the user using the ARL13B (red) channel. Based in these two 3D points, the Simple Neurite Tracer (SNT) plugin^54^ was used in FIJI, using default parameters, to build a simple path (curve) between them. This curve in the volume was find using two directional searches based on a cost defined by the selected points and considering the intensities distribution of the image and a measure of ‘tubeness’ at the points. This curve defined in the red channel was then manually validated in the green (acetylated α-tubulin) channel where its end points were eventually adjusted and the curve updated. The 3D curves for every cilia were projected to the 2D along with the transformation of the Z-stack in an image using FIJI’s maximum intensity Z-projection tool. 2D curves were divided in ten segments of similar distances, and each segment was thickened to about three pixels wide defining ten regions of interest covering each cilium.

### Electron microscopy

For scanning electron microscopy (SEM) analysis, cells were grown on glass coverslips, fixed overnight in a mixture of 4% paraformaldehyde and 3,5% glutaraldheyde in 0.1 M phosphate buffer saline pH 7,2 (PBS) at 4 °C. After that, cells were washed in PBS, dehydrated through a series of ethanol solutions of increasing concentration and submitted to critical point drying with CO_2_ in a DPC-1 Denton Vacuum Critical Point Drying apparatus. Dried samples were affixed to aluminum stubs with carbon tape and sputter-coated with gold in DeskII Denton Vacuum for 120 seconds. Samples were analyzed in high vacuum in a Jeol-5900 LV SEM operated at an accelerating voltage of 20 kV.

### Generation of CRISPR/Cas9 CCDC28B cell lines

We designed five gRNAs using the online design tool at http://crispr.mit.edu and cloned them into the pLentiCRISPRv2 lentiviral vector (a gift from Dr. Feng Zhang, Addgene plasmid #52961) following Zhang’s laboratory protocol^35,36^. To obtain lentivirus we co-transfected Hek293 cells plated in 10 cm dishes with the pLentiCRISPRv2 construct and the packaging vectors pCMV-VSV-G (Addgene #8454) and psPAX2 (Addgene #12260). The supernatant containing the virus was collected 48 hours after transfection, centrifuged a 4000 RPM for 15 min, passed through a 0.45μm filter and stored at −80 °C until used. hTERT-RPE cells were transduced at 60% confluency in 6-well dishes with virus-containing supernatant diluted 1/2 with fresh medium in the presence of 8 μg/ml Polybrene (Sigma). Selection was started 24 hours after transduction supplementing media with 10 μg/ml puromycin. For cell sorting and single cell deposition, hTERT-RPE cells were grown and transduced in a 10 cm dish. After 5 days under puromycin selection (6 days post-transduction), cells were resuspended and sorted into a 96-well plate using a BD FACSAria^TM^ Fusion (BD Biosciences). To identify the mutations in *CCDC28B*, genomic DNA was obtained by standard methods, exon 2 was amplified by PCR, PCR products were cloned into pGEM-T-easy following the manufacturer’s instructions (Promega) and individual constructs were purified and sequenced. For clone B1, cDNA was generated using SuperScript® III First-Strand Synthesis kit (Invitrogen) with oligodT primers. *CCDC28B* was amplified with specific primers, PCR products were cloned into pGEM-T-Easy and sequenced. The sequences of all primers and gRNAs used in this study are available upon request.

### Zebrafish: morpholino knockdown

To target *ccdc28b* in zebrafish we used our previously validated splice-blocking morpholino^14^. MOs were obtained from Gene Tools. We injected TAB5 wild-type zebrafish embryos at a one to two-cell stage with 0,5 nanoliters of MO solutions prepared to deliver 5 ng of MO. mRNA for rescue experiments was prepared using the *ccdc28b* wt and *ccdc28bΔ*4–10 ORFs cloned into pCS2+ and the Ambion mMessage mMachine SP6 mRNA kit. Experiments in zebrafish were performed following protocol 010–13 approved by the Institut Pasteur de Montevideo ethics committee for the use of animal models.

### Zebrafish: CRISPR/Cas9 genome editing

CRISPR/Cas9 was used to generate mutations in the coding region of zebrafish *ccdc28b* following the methods described in^55^. Four gRNAs were designed using CRISPRscan tool. gRNAs were produced as described^55^. In short, a DNA template containing T7 promoter, the sgRNA targeting sequence and sgRNA invariable 3′end was generated by fill in PCR. This template was purified and used for *in vitro* transcription using Ambion MAXIscript SP6/T7 *In Vitro* Transcription Kit (Thermo Fisher Scientific). After DNAse treatment, sgRNAs were purified through precipitation with sodium acetate and ethanol. All gRNAs were tested and then two of them targeting exon 2 were selected for the study. All sequences and estimated efficiencies are available upon request. Cas9 mRNA was synthetized *in vitro* from plasmid pT3TS-nCas9n^56^ which codifies zebrafish codon-optimized Cas9 fused to nuclear localization signals, using the mMESSAGE mMACHINE Tanscription Kit (Thermo Fisher Scientific). zf-nCas9n mRNA was purified using MicroSpin G-50 columns (GE Healthcare). Zebrafish embryos in one cell-stage were injected in the cell cytoplasm with a combination of 50 pg of each sgRNA and 100 pg of zf-nCas9n mRNA. General embryo morphology and the aspect of cilia in different organs were analyzed at 48 hpf.

### Statistical analyses

The proportion of cilia positive cells was calculated by counting the number of cilia with clear acetylated-tubulin signal over the total number of cells and results are expressed as a proportion with a 95% confidence interval (CI). The comparison between the different samples was performed as described^57^, using a test of hypothesis specific for comparison of two proportions (hypothesis test for proportions). For the analysis of cilia length, we first tested the sets of data for normal distribution, using the Shapiro-Wilk test, and variance homogeneity using the Levene test. When necessary, data were Ln transformed in order to meet the distributional assumptions required by the statistical tests. To compare three or more groups with normal distribution we used one-way ANOVA. In the case of datasets without normal distribution we used non-parametric tests, the two-tailed Mann Whitney in order to compare two sets of data or the Kruskal Wallis to compare more than two sets. To compare the distribution of classes in the zebrafish experiments we used Chi-squared test (χ^2^) when data was pooled and both Wilcoxon rank test and paired t-test for the analysis of percentage of embryos per class and condition. The tests used are appropriately indicated in the corresponding figure legends.

## Electronic supplementary material

Supplementary Information
