# Supplementary material for: Kinesin 1 regulates cilia length through an interaction with the Bardet-Biedl syndrome related protein CCDC28B
Source: Sci Rep. 2018 Feb 14;8:3019. doi: 10.1038/s41598-018-21329-6 (PMC5813027; doi:10.1038/s41598-018-21329-6)
Supplement: Supplementary file 1 — Supplementary Information [file 41598_2018_21329_MOESM1_ESM.pdf]

Supplementary Information

Kinesin 1 regulates cilia length through an interaction with the Bardet-Biedl syndrome related protein CCDC28B.

Rossina Novas[1], Magdalena Cardenas-Rodriguez[1], Paola Lepanto[1], Matías Fabregat[1], Magela Rodao[2], María Inés Fariello[3, 4], Mauricio Ramos[5], Camila Davison[2], Gabriela Casanova[2], Lucía Alfaya[6], Federico Lecumberry[3,5], Gualberto González-Sapienza[6], Florencia Irigoín[7,1] and Jose L. Badano[1,*]

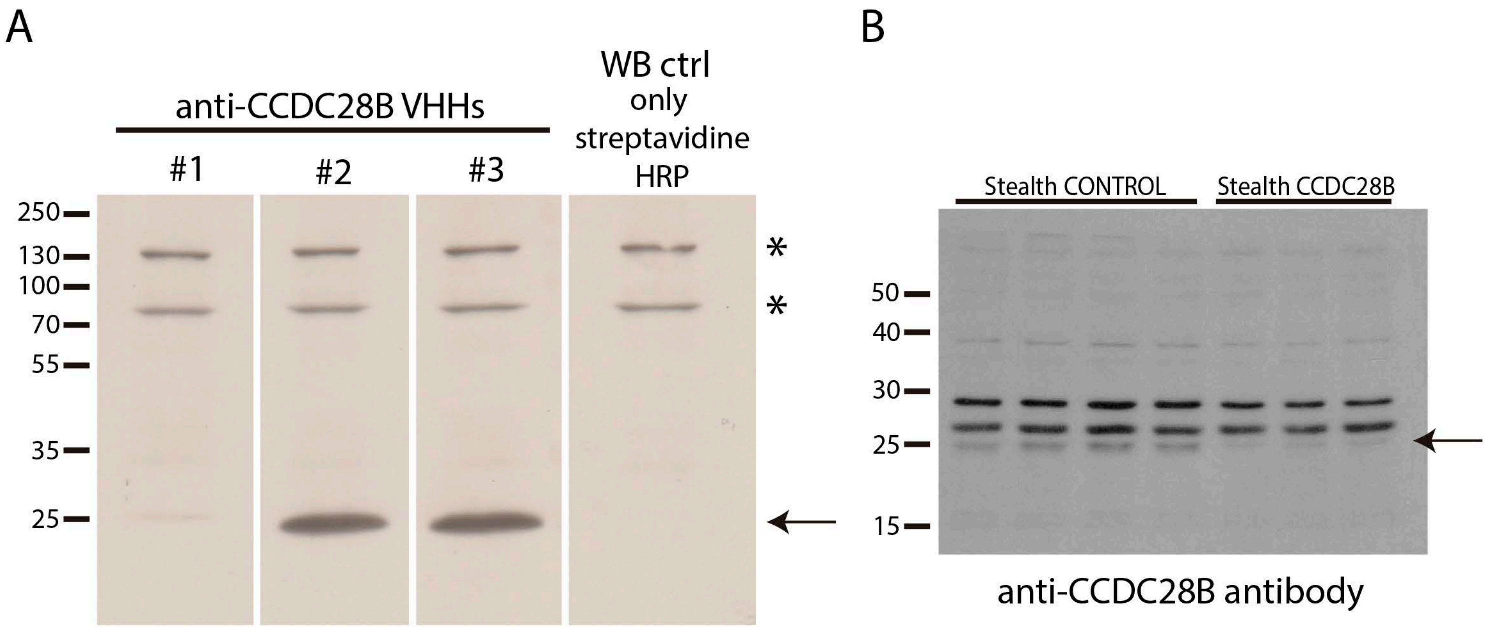

Figure S1: CCDC28B antibodies. A) Evaluation of different CCDC28B single domain llama antibodies (VHH) by western blot. Clones #2 and #3 recognize a band in Hek293 cell lysates where CCDC28B was overexpressed. Streptavidin-HRP alone was used as control and detects two non-specific bands of high molecular weight (*). **B)** Our rabbit polyclonal anti-CCDC28B antibody (Genzyme) recognizes CCDC28B (which is knockdown by our stealth CCDC28B oligo) as well as other non-specific bands.

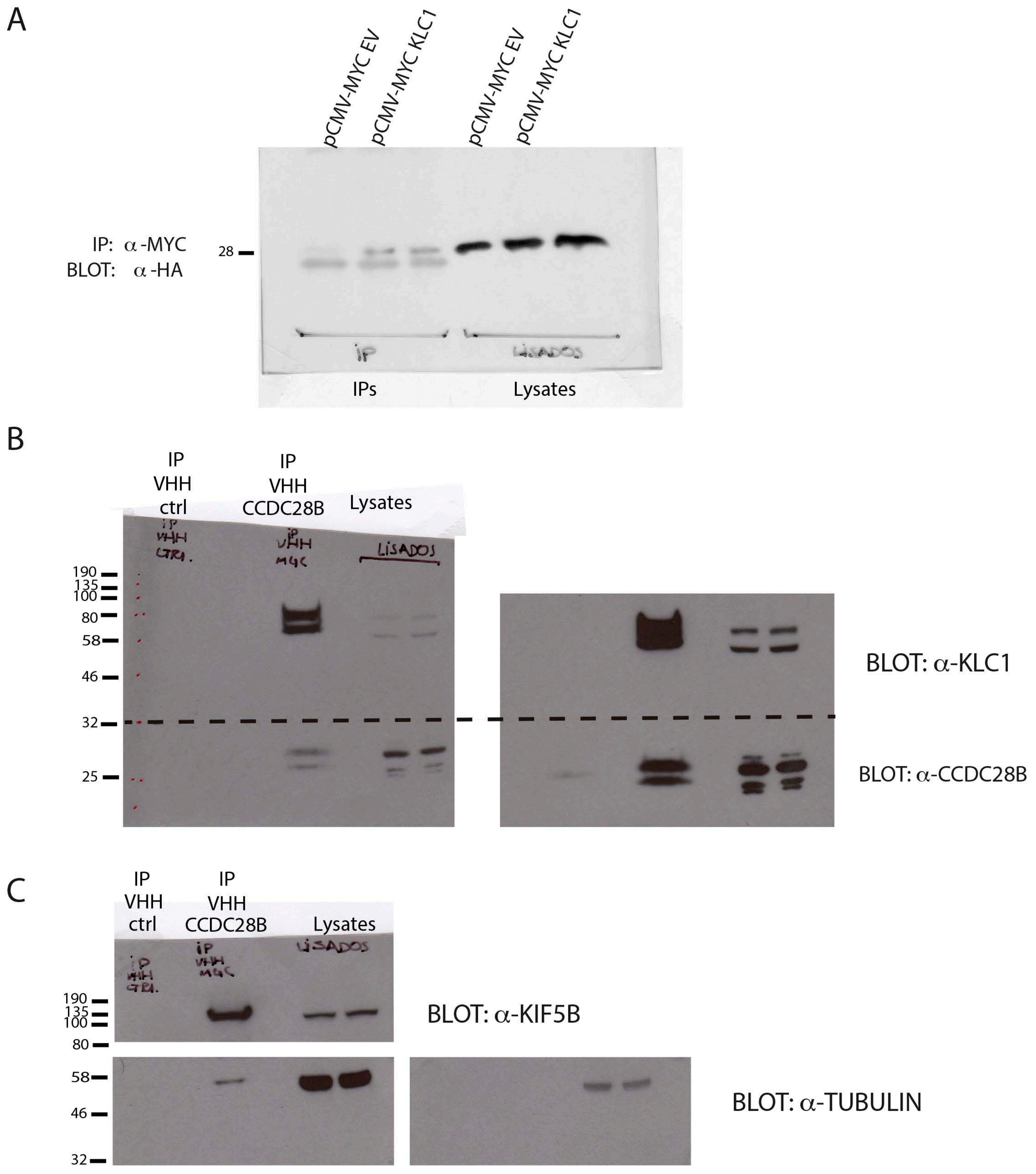

Figure S2: Full-length blots corresponding to figure 1. A) Full length blot corresponding to figure 1B. **B-C)** Full-length blots corresponding to figure 1D. **B)** The blot was cutted in two at the 32 KDa ladder band (marked by the dashed line). The two membrane pieces were aligned before exposure. Two exposures are shown.**C)** The upper membrane from B was stripped, cutted at the 80 KDa marker and re-probed with anti-KIF5B and anti-Tubulin antibodies (two exposures of tubulin are shown).

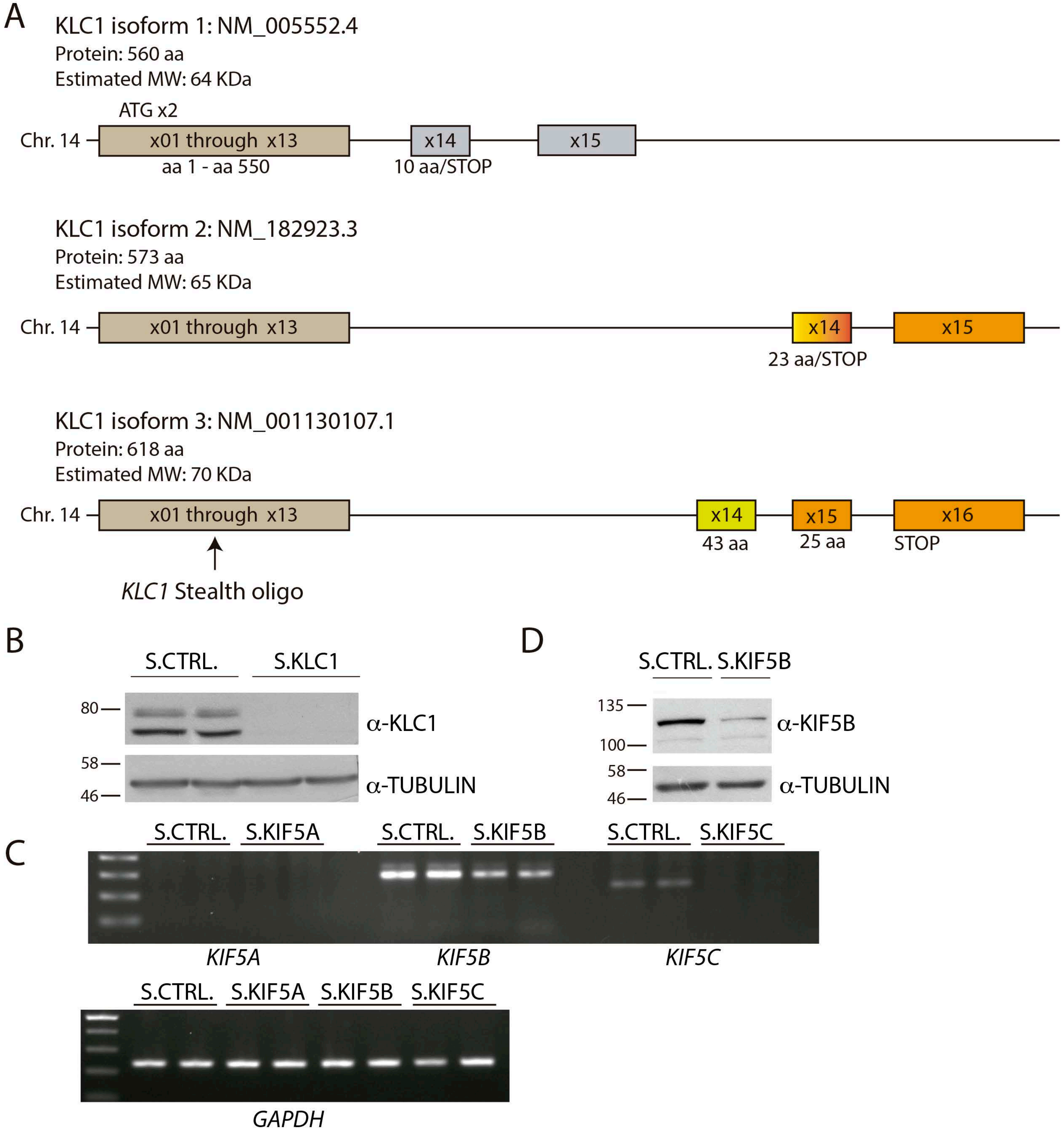

Figure S3: KLC1 isoforms and validation of stealth RNA oligos. A) Schematic representation of the three human KLC1 isoforms highlighting the position of the stealth RNA oligo that was used in this work. **B)** Western blot showing a reduction of KLC1 levels upon transfection of the stealth RNA oligo (S.KLC1) in hTERT-RPE cells. Full-length gels are shown in Fig. S11. **C)** Semi-quantitative RT-PCR analysis in hTERT-RPE cells showing a reduction in KIF5B and KIF5C mRNA levels upon transfection of their respective stealth ARN oligos. The expression of KIF5A is low in this cell line. GAPDH was used as control. Full gels are shown in Fig. S11. **D)** Western blot confirming knockdown of KIF5B in cells transfected with the corresponding RNA oligo (S.KIF5B). Tubulin was used as control. Full-length blot shown in Fig. S11.

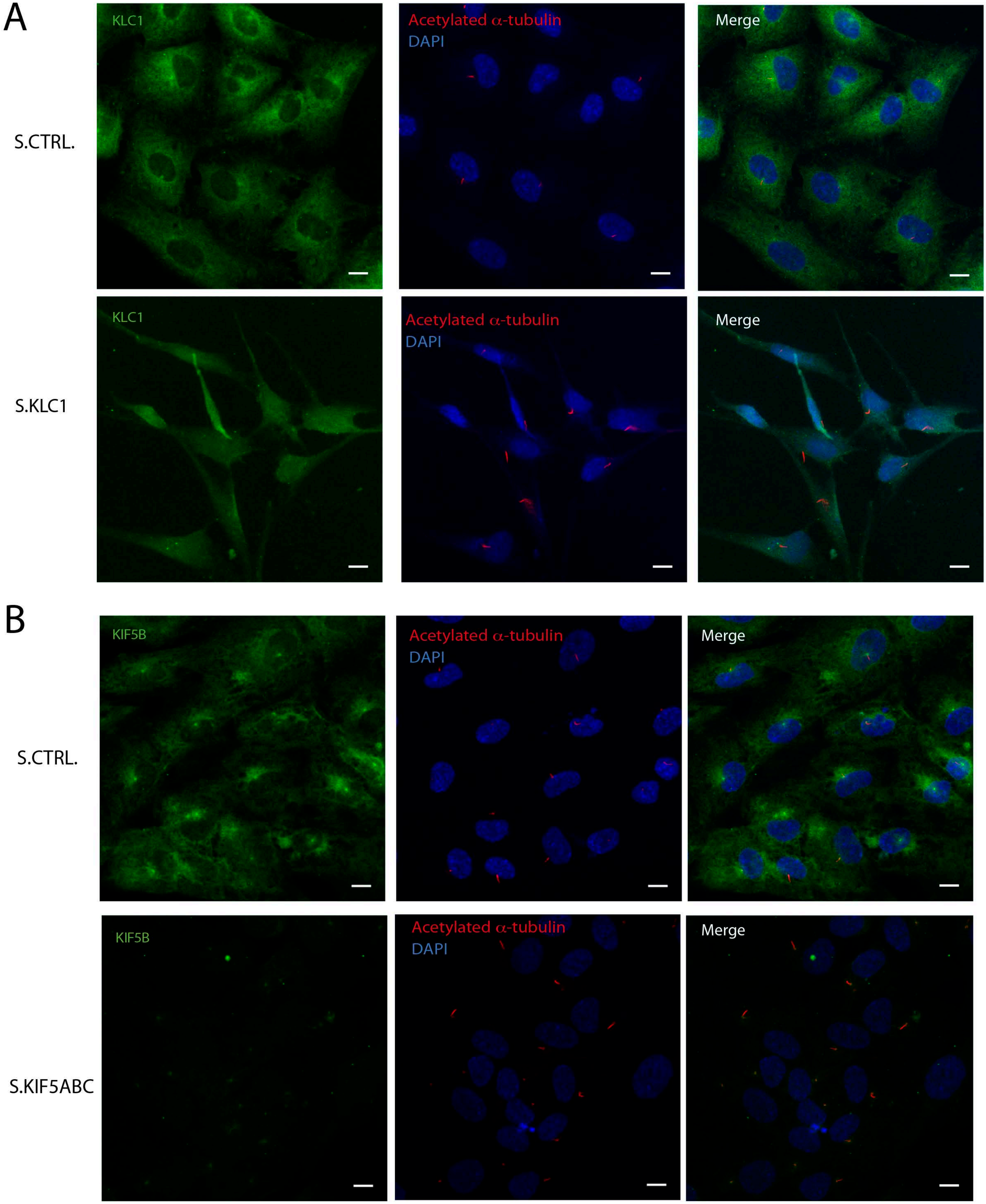

Figure S4: Validation of the anti-KLC1 and KIF5B localization. A) Endogenous KLC1 localizes in the cytoplasm and pericentriolar region and the signal is lost upon transfection with the specific RNA oligo (S.KLC1). **B)** Similarly, the localization of KIF5B to the pericentriolar region is specific.

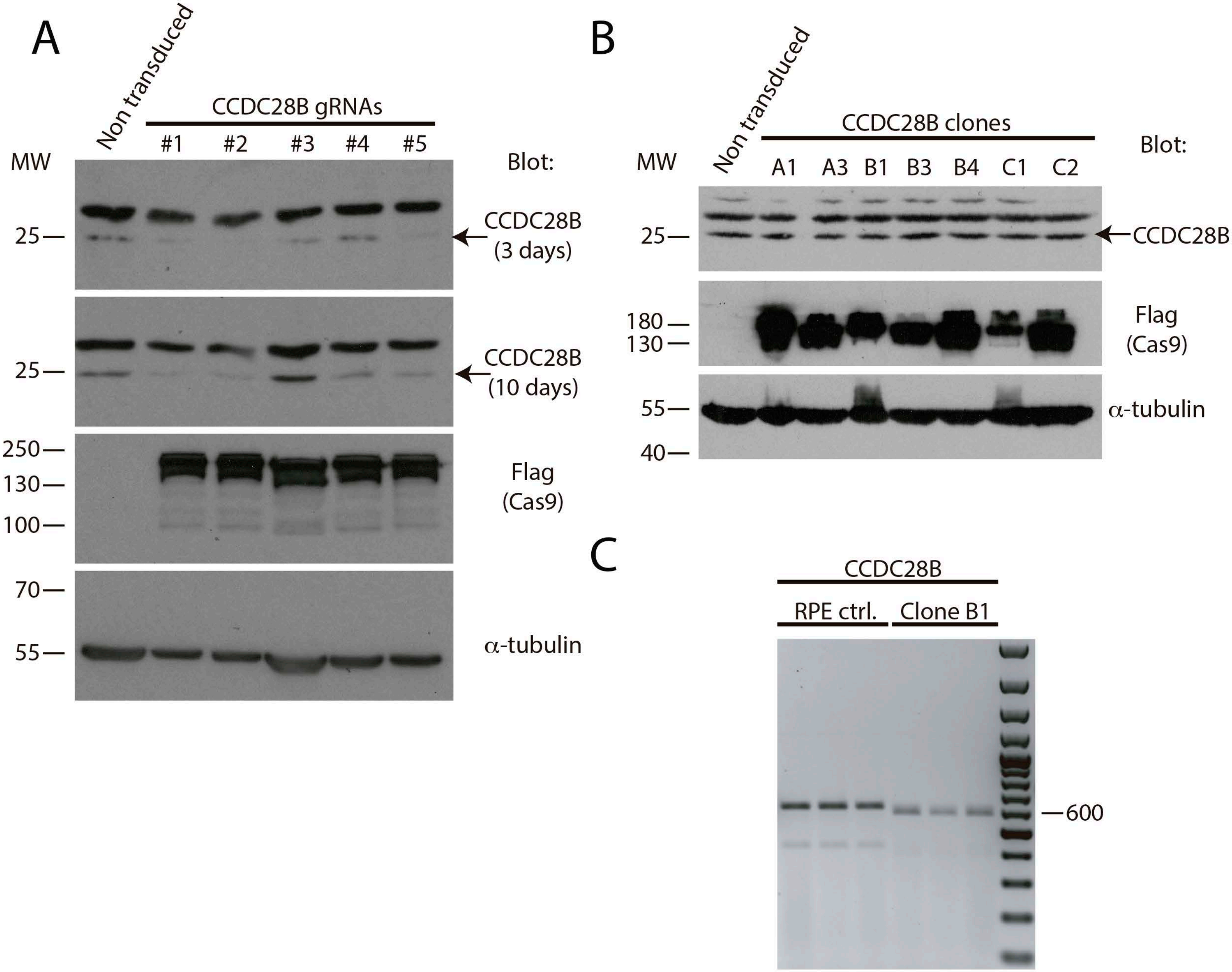

Figure S5: Generation of knockout CCDC28B hTERT-RPE cells with CRISPR/Cas9. A) Western blot analysis of hTERT-RPE cells transduced with pLentiCRISPRv2 targeting CCDC28B. Five different gRNAs were tested. In most cases, a reduction in CCDC28B levels is observed at 10 days after transfection but is some cases levels of protein began to recover compared to day 3. The expression of Flag-tagged Cas9 was observed with an anti-Flag antibody. Tubulin was used as loading control for day 10 blot. **B)** Clones obtained after sorting and single cell deposition in 96 well plate were analyzed by western blot with anti-CCDC28B antibody, anti-Flag and anti-tubulin. In all cases, CCDC28B is still detected. Membranes were cut in three as shown. **C)** Semi-quantitative RT-PCR showing amplification of the *CCDC28B* open reading frame from hTERT-RPE control cells and clone B1 cells.

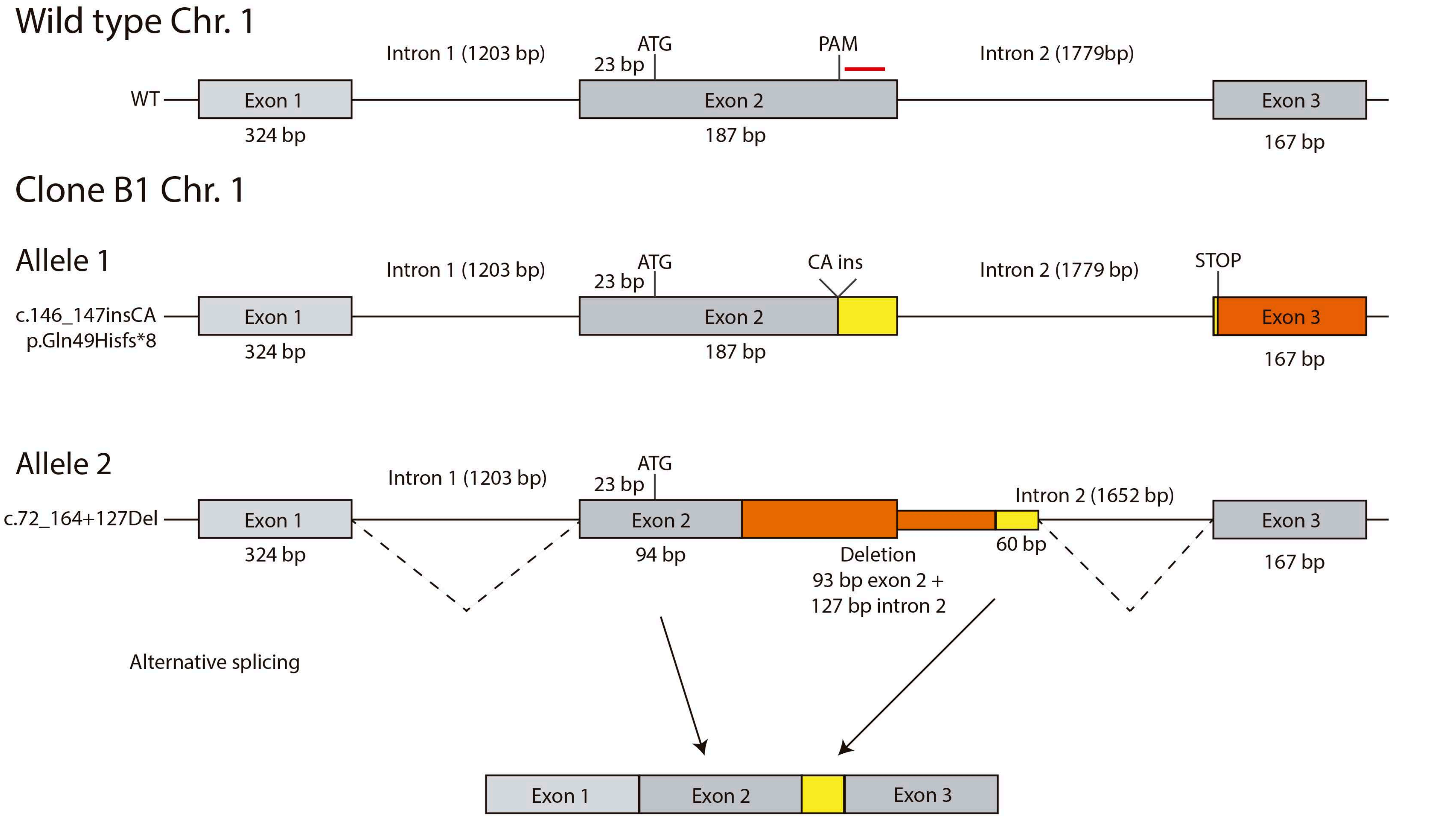

Figure S6: Partial representation of the CCDC28B genomic region. Exons 1 to 3 (from a total of 6) are shown and the position of gRNA#1 is highlighted with a red line. Schematic representation of the two CCDC28B alleles in the hTERT-RPE CRISPR CCDC28B clone B1. One allele carries a two base insertion (CA) generating a frameshift an premature stop codon. The second allele presents a 220 bp deletion eliminating the last 93 bases of exon 2 and the first 127 bases of intron 2. Sequencing cDNA showed that 60 bases of the remnant intron 2 (yellow) are incorporated into exon 2 thus coding for 20 novel residues that are in frame with the wild type protein encoded from exon 3 onwards.

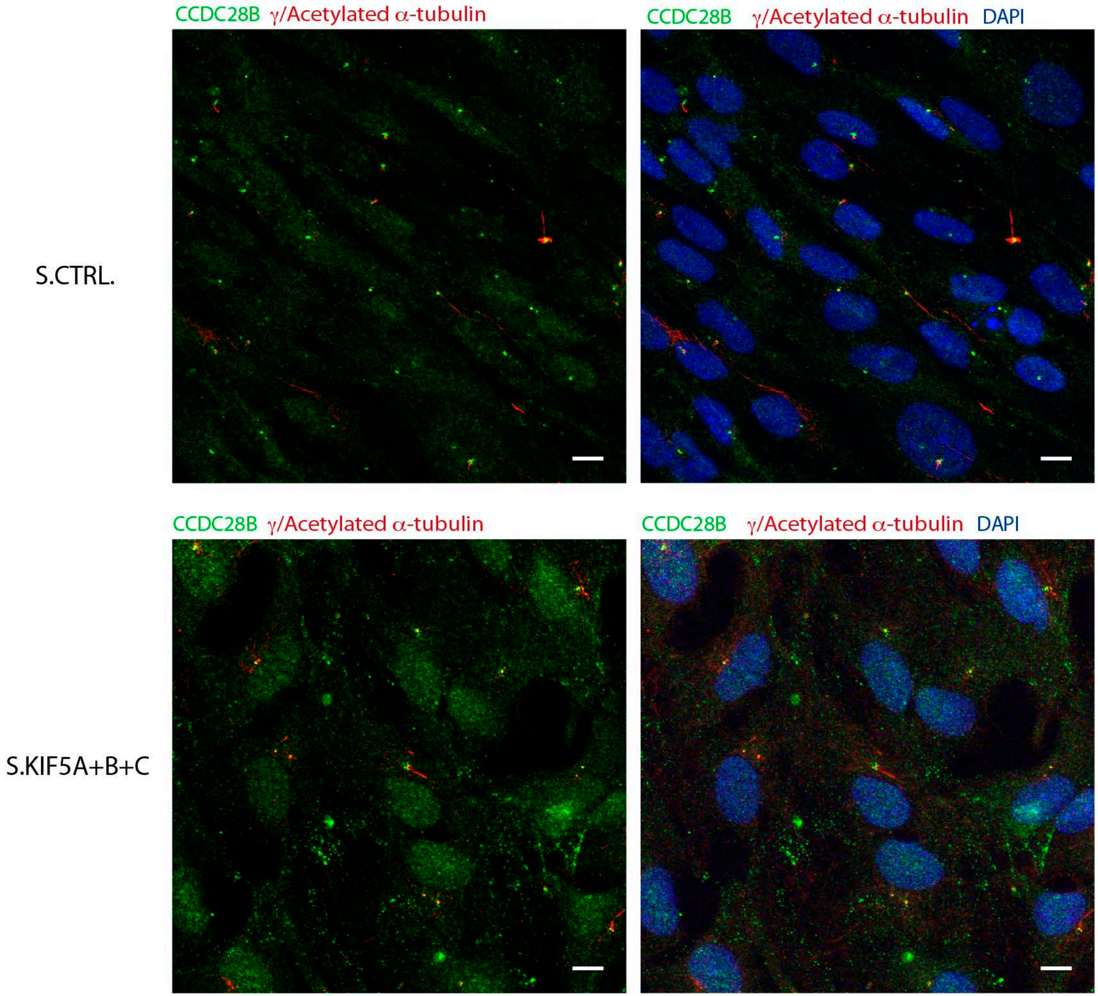

Figure S7: Evaluation of the CCDC28B nuclear accumulation in KIF5 KD cells. CCDC28B accumulates in the nucleus in KIF5 KD cells (targeting all three KIF5s: KIF5A+B+C).

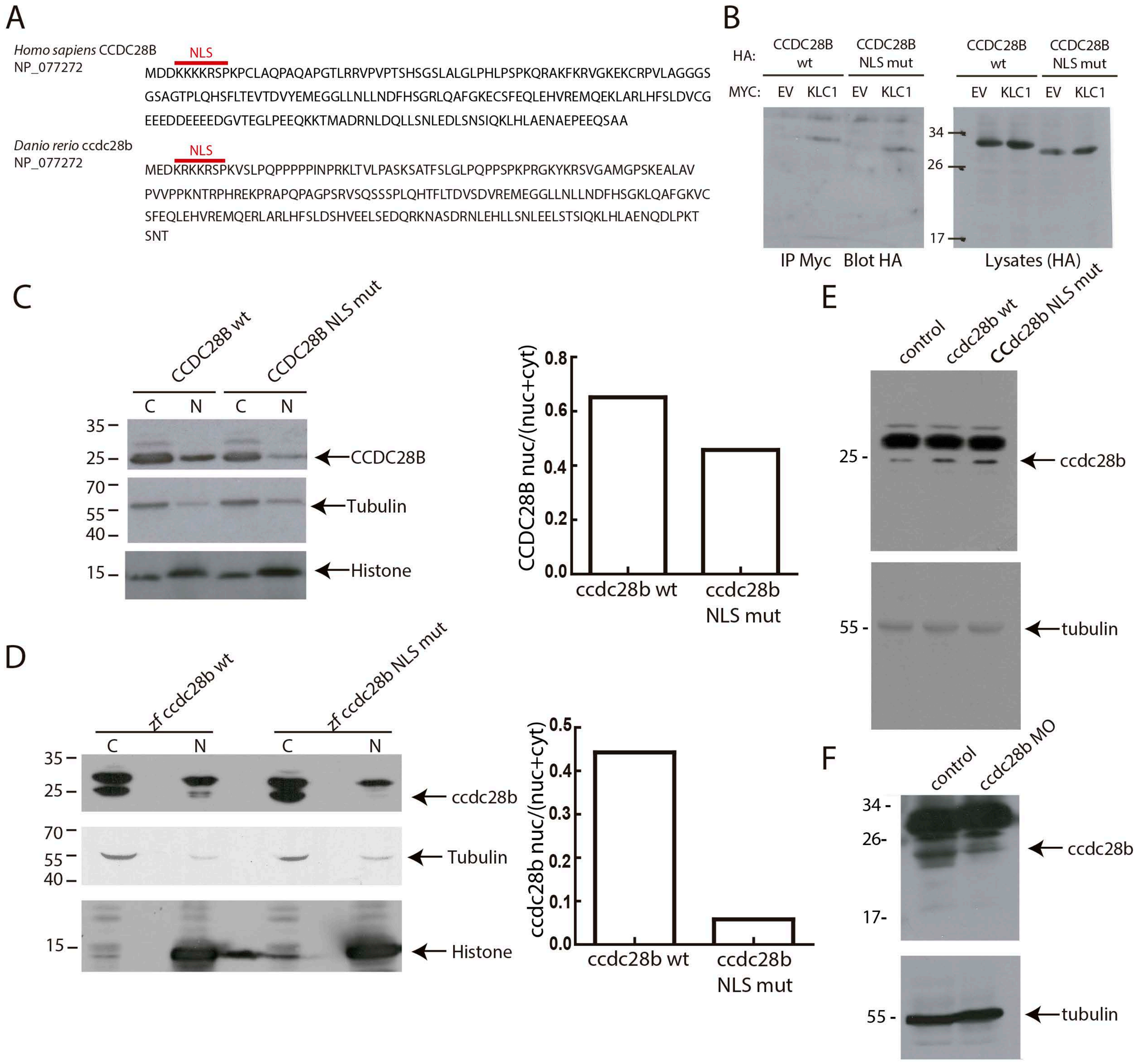

Figure S8: Evaluation of the CCDC28B NLS. A) The protein sequences of human and zebrafish CCDC28B are shown highlighting the position of a predicted nuclear localization signal (NLS). **B)** Co-immunoprecipitation assay showing that the human CCDC28B NLS mut can also be immunoprecipitated by Myc-KLC1. **C)** Cell fractionation of cells transfected with wt or mutant CCDC28B expressing constructs. **D)** Cell fractionation assay using hTERT-RPE cells overexpressing zebrafish ccdc28b, both wild type (wt) and NLS mut (Δ4-10). The membranes were cut between 35 and 40 KDa in C and at 35 Kda in D. The lower molecular weight part was probed for CCDC28B, then stripped and probed for histone. Full-length blots are shown in Fig. S12. **C and D)** Quantification of the nuclear/total (nuclear+cytoplasmic) ratio using the blots shown, normalizing with tubulin to reduce the impact of cytosolic contamination in the nuclear fractions. **E)** Wild type or NLS mutant zebrafish ccdc28b mRNA was injected in 2-4 cell zebrafish embryos and total protein was extracted at 48 hours (a pool of 30 embryos was used for each condition). 12% gel was used. **F)** ccdc28b levels are reduced in MO injected embryos. 15% gel was used. Non-injected embryos were used as control in both E and F.

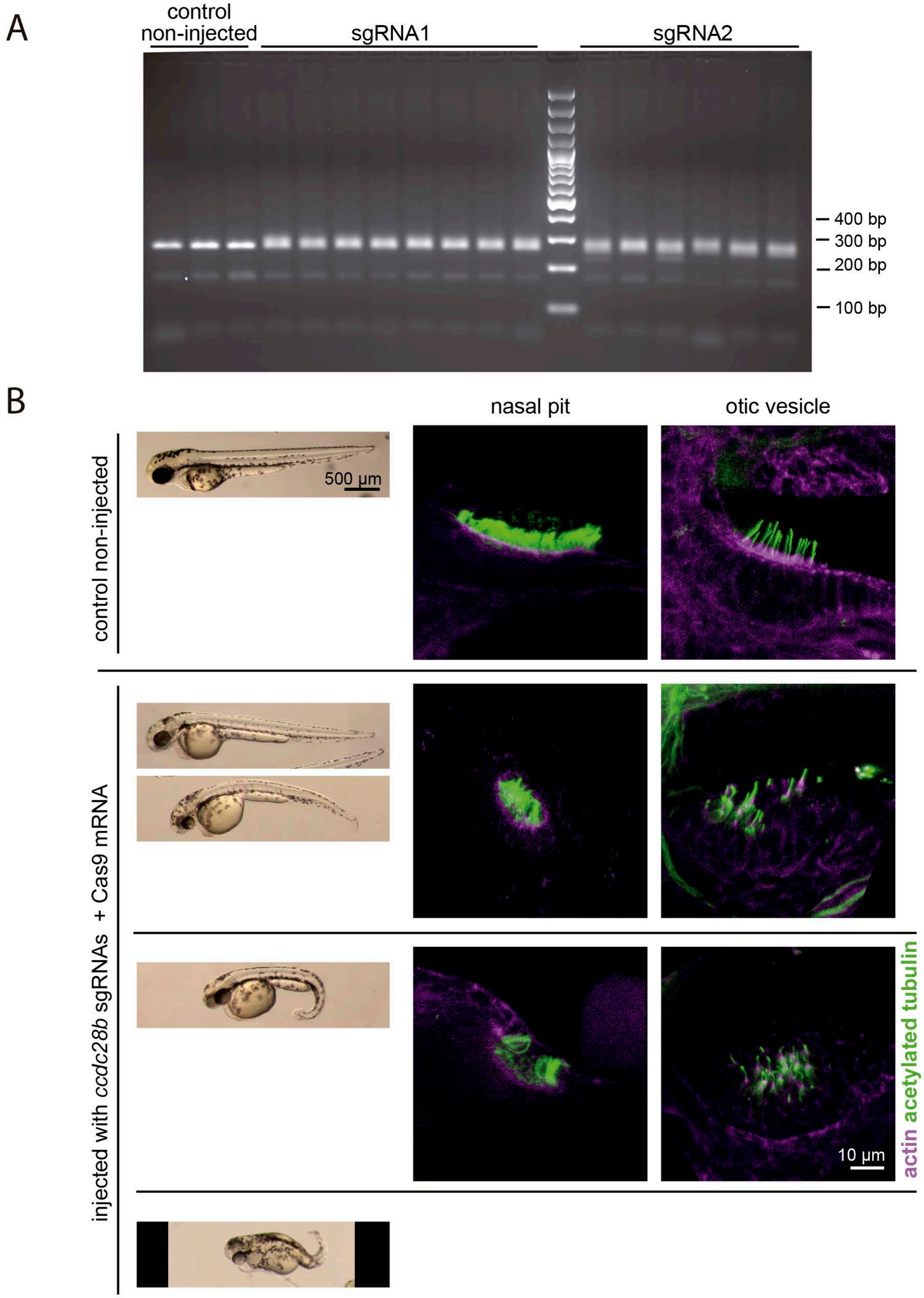

Figure S9: *ccdc28b* CRISPR/Cas9 F0 zebrafish. A) PCR DNA analysis of individual embryos injected with two *ccdc28b* sgRNAs. In both cases the PCR result is compatible with the gRNAs driving genomic changes in the locus. **B)** CRISPR/Cas9 genome editing of *ccdc28b* generated embryonic phenotypes similar to those observed in embryos injected with *ccdc28b* morpholinos. The left column shows low magnification images of the general morphology of embryos injected with the combination of sgRNA and zf-nCas9n mRNA at 48 hpf. Phenotypes resemble those found in embryos injected with morpholinos. Embryos with similar phenotypes were fixed to analyze the aspect of cilia in different organs through immunofluorescence. Actin and cilia axoneme were stained with TMR conjugated phalloidin (magenta) and anti-acetylated tubulin respectively (green). As in embryos injected with morpholinos, embryos injected with sgRNA targeting *ccdc28b* showed perturbed ciliated tissues.

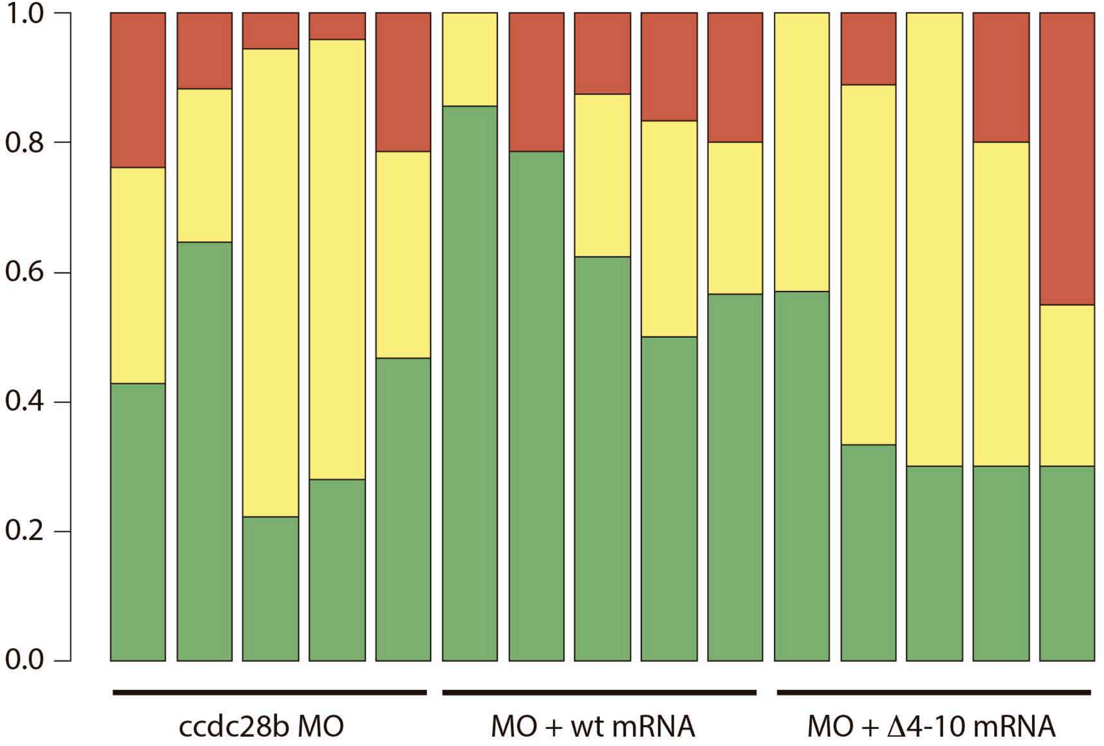

Figure S10: Individual rescue experiments in zebrafish. Barplots showing the proportion of zebrafish phenotypic classes in the five independent injections that were used in the analysis shown in Fig. 6C. Class A = green; Class B =yellow; Class C = red.

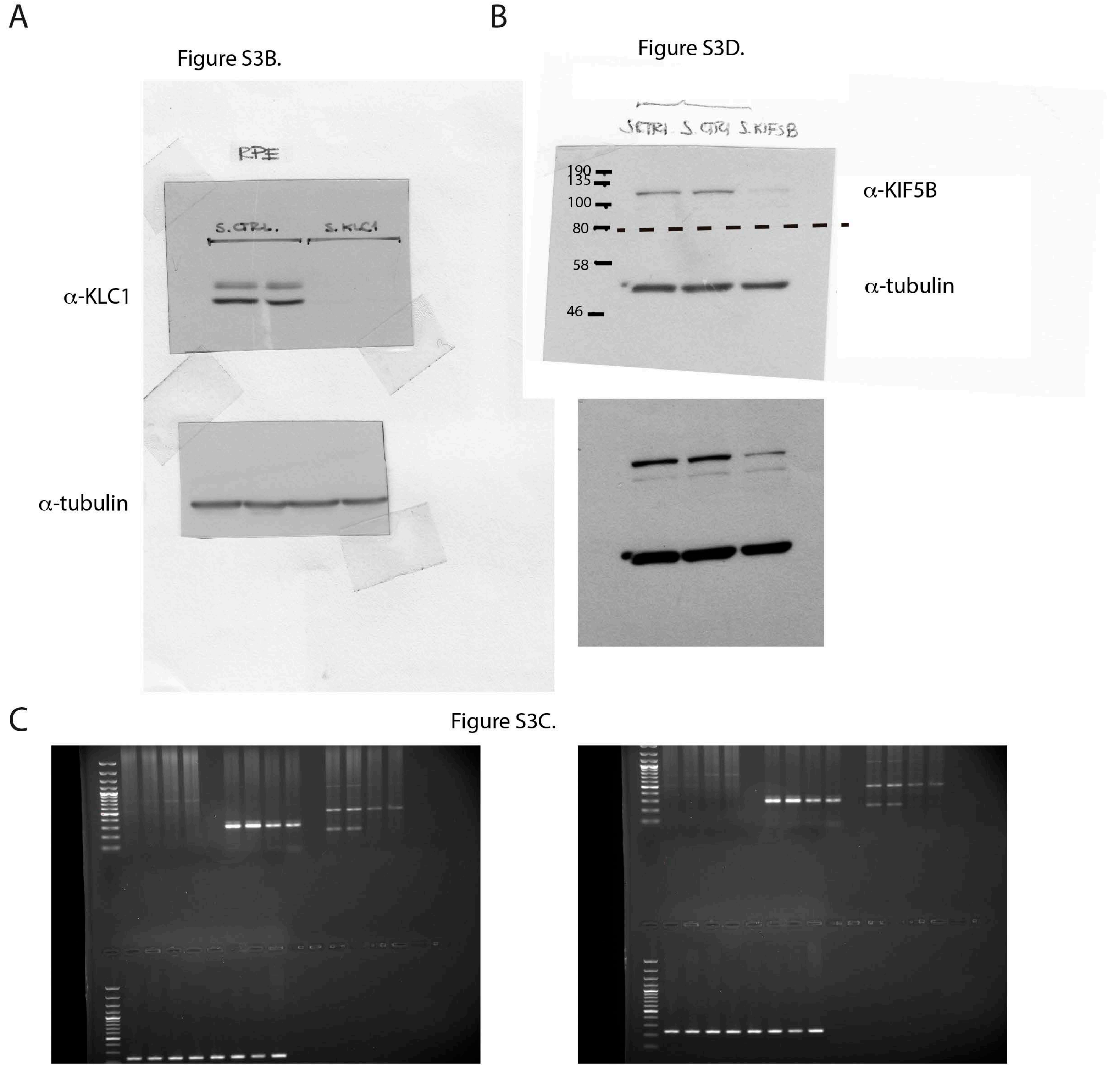

Figure S11: Full-length blots corresponding to figure S3. A) Blots corresponding to Fig. S3B. **B)** Blots corresponding to Fig. S3D. The blot was cutted in two at the 80 KDa ladder band (marked by the dashed line). The two membrane pieces were aligned before exposure. **C)** Full length gels presented in Fig. S3C.

Gels corresponding to Fig. S8 C

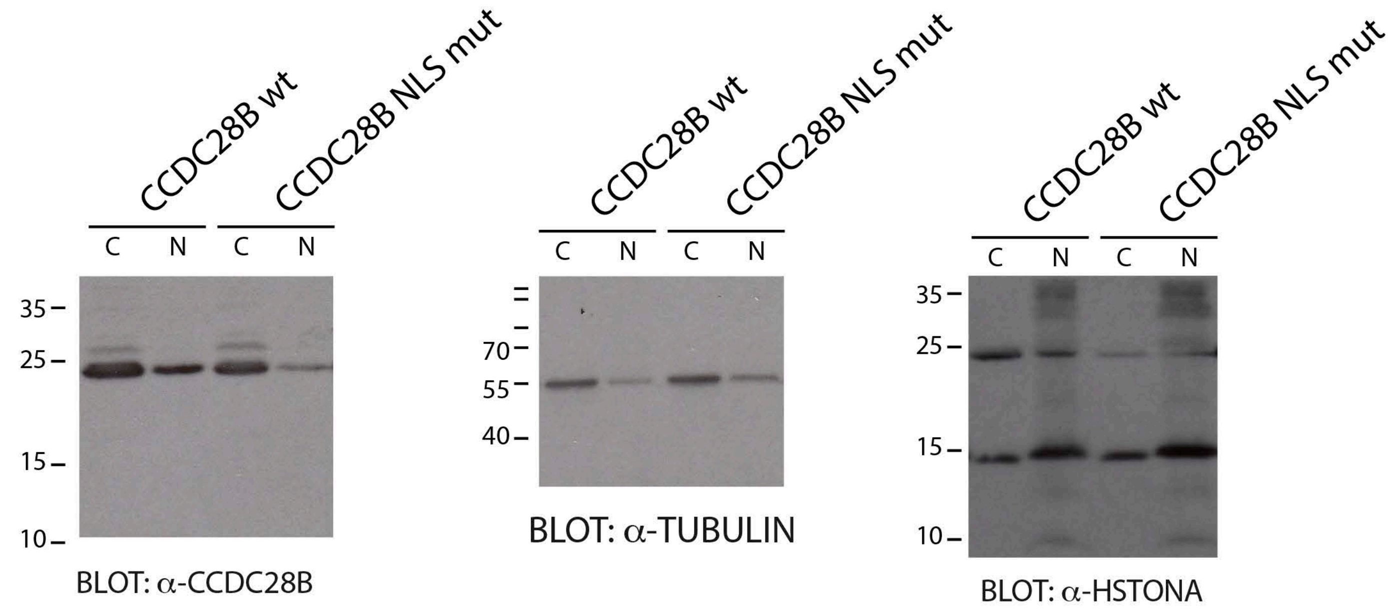

Gels corresponding to Fig. S8D

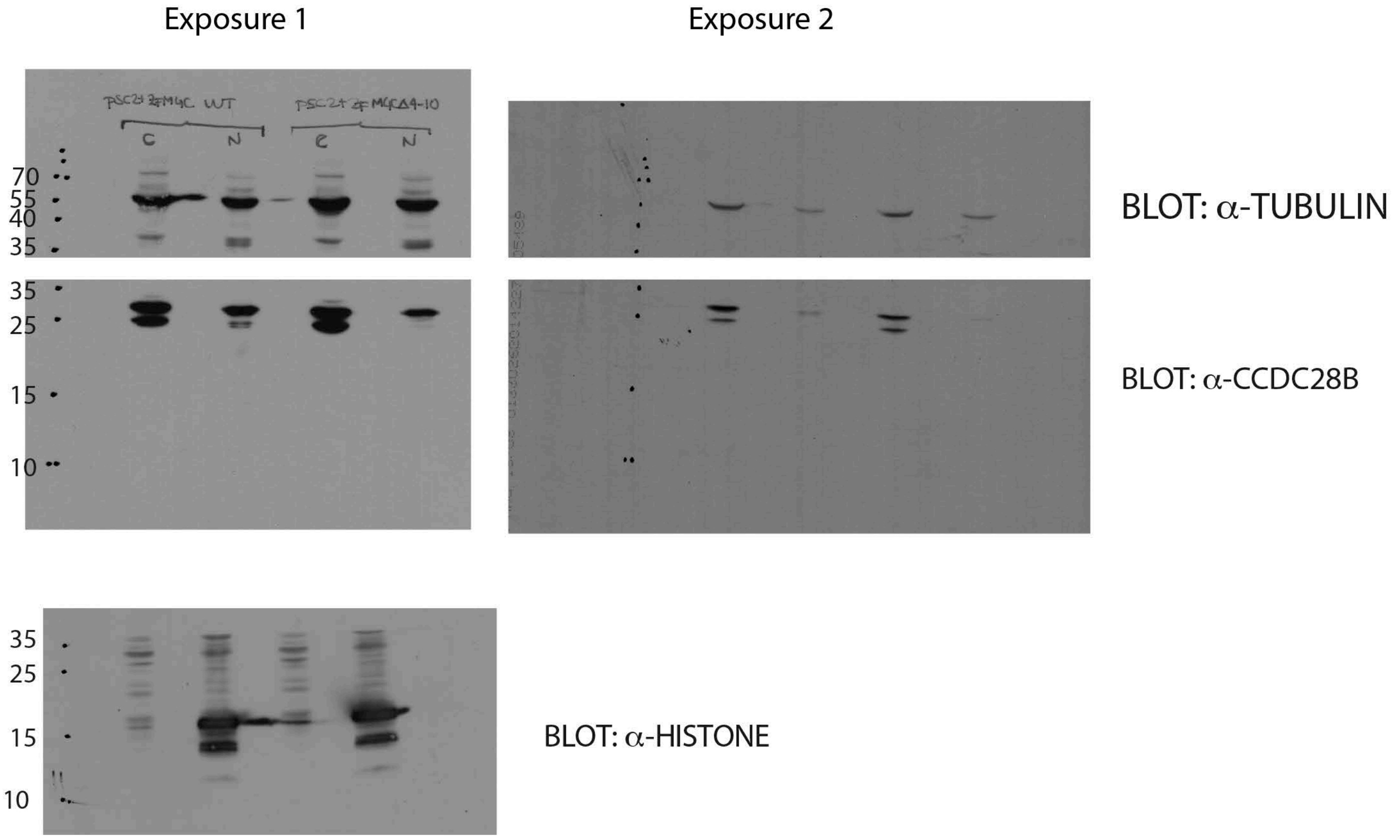

Figure S12: Full-length blots corresponding to figure S8.

List of proteins identified by MALDI TOF/TOF MS from bands

Label	Description	Accession N^0	Sequence coverage (%)	Mascot protein *score*	# of assigned peptides *
50KDa	α-Tubulin	gi|340021	43	264	14
50KDa	β-tubulin	gi|18088719	52	409	23
120KDa	kinesin-1 heavy chain 5B	gi|4758648	52	591	47
25KDa	Coiled-coil domain-containing protein 28B	gi|110349769	45	237	8

* at least three sequences were confirmed by MS/MS analysis
